# Genome-guided discovery of antibiotic activity in *Streptomyces virginiae* THA-960 against multidrug resistant *Staphylococcus aureus*

**DOI:** 10.1016/j.isci.2026.116311

**Published:** 2026-06-09

**Authors:** Trang Thi Minh Nguyen, Jeyong Jung, Xiangji Jin, Qiwen Zheng, Jaeyoung Choi, Tae-Hoo Yi

**Affiliations:** 1 Graduate School of Biotechnology, Kyung Hee University, Yongin 17104, Republic of Korea; 2 Department of Dermatology, School of Medicine, Kyung Hee University, 23 Kyungheedae-ro, Dong-daemun, Seoul, Republic of Korea; 3 Department of Convergent Biotechnology and Advanced Materials Science, College of Life Sciences, Kyung Hee University, Yongin 17104, Republic of Korea; 4 BK21 Interdisciplinary Program in IT-Bio Convergence System, Kyung Hee University, Yongin 17104, Republic of Korea

**Keywords:** health sciences, medical microbiology, microbial genomics

## Abstract

The emergence of multidrug-resistant *Staphylococcus aureus* (MDR-*S. aureus*) demands innovative strategies to identify robust microbial producers of potent antibiotics. This study characterizes *Streptomyces virginiae* THA-960, a soil-derived actinomycete, as a producer of the known anti-MDR-*S. aureus* antibiotic. Phenotypic screening showed THA-960’s efficacy against clinical MDR-*S. aureus* isolates, with MICs as low as 0.08 mg/mL, outperforming conventional antibiotic-producing *Streptomyces*. Time-kill assays and SEM confirmed rapid bactericidal action via cell wall disruption. Complete genome sequencing revealed a rich biosynthetic potential, housing 31 specialized metabolite gene clusters. Phylogenomic analysis of 521 *Streptomyces* genomes delineated *S. virginiae* into distinct groups and showed that the amycomicin biosynthetic gene cluster is conserved within a specific taxonomic group, providing a genomic roadmap for targeted strain selection. Crucially, Ultra-performance liquid chromatography coupled with quadrupole time-of-flight mass spectrometry (UPLC-Qtof-MS) analysis detected a metabolite feature putatively assigned as amycomicin in THA-960. This integrated multi-omics study provides a phylogenomic framework for the targeted exploitation of *Streptomyces* strains against antimicrobial resistance.

## Highlights

• First complete genome of *S. virginiae* reveals rich biosynthetic potential for antibiotics
• Integrated multi-omics approach validates potent antibiotic activity via cell wall disruption
• Amycomicin-producing *S. virginiae* THA-960 combats multidrug-resistant *S. aureus*
• Amycomicin BGC conserved in a specific genomic group enables targeted discovery

## Graphical abstract

**Figure:**
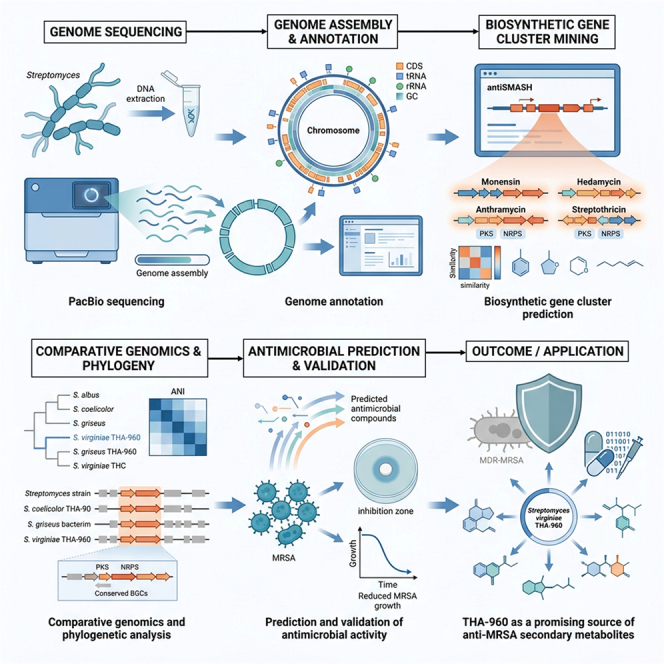

## Introduction

The rapid rise of antimicrobial resistance remains a critical global health concern, with projections indicating that annual mortality could reach 10 million deaths by 2050 without new therapeutic interventions.^1^ Among drug-resistant pathogens, multidrug-resistant *Staphylococcus aureus* (MDR-*S. aureus*) is particularly concerning, as it is a well-recognized cause of diverse infections in both humans and animals. MDR-*S. aureus* is implicated in subcutaneous and deep-tissue abscesses, skin infections such as cellulitis and impetigo, as well as osteomyelitis, septicemia, and meningitis,^1^^,^^2^ and accounts for more than one million infections and over 120,000 deaths worldwide each year.^1^^,^^3^^,^^4^^,^^5^ Resistance mechanisms in MDR-*S. aureus* continue to erode the efficacy of β-lactams and increasingly compromise glycopeptides, lipopeptides, and several next-generation agents, underscoring the urgent need for therapeutics with novel modes of action.^6^ *S. aureus* strains, exhibiting multidrug-resistance against nearly all therapeutic agents, have emerged as a significant challenge not only in hospital-acquired but also in community-acquired infections, making MDR-*S. aureus* a major concern in both settings.^2^ Therefore, there is an emphasis on the necessity for the discovery of novel antibiotics capable of overcoming resistance in existing MDR-*S. aureus* strains and effectively operating in diverse environments.

*Streptomyces* has been recognized as the most prominent group of bacteria in the soil ecosystem.^7^ The genus has historically served as the predominant source of clinically valuable antibiotics, accounting for approximately 70%–80% of approved anti-infective agents.^8^ However, traditional bioassay-guided screening has become increasingly limited due to rediscovery of known compounds.^9^ Genomic analyses indicate that Streptomyces species possess substantial untapped biosynthetic capacity: most genomes harbor 36–48 biosynthetic gene clusters (BGCs), only a minority of which have been linked to characterized metabolites.^10^ Recent advances in genome mining and BGC-activity prediction have revitalized natural product discovery by enabling targeted exploration of phylogenetically distinct strains and silent metabolic pathways. *Streptomyces* are studied to produce indolmycin, tubercidin, leupeptin, and multiple other compounds, underscoring the genus’s rich antibacterial potential.^11^^,^^12^^,^^13^

Recent genome-driven discovery efforts have identified amycomicin, a nanomolar-potency antibiotic with a minimum inhibitory concentration (MIC) at 30 nM, that selectively inhibits β-ketoacyl-acyl carrier protein synthase III (FabH) in *S. aureus* and retains activity against multidrug-resistant strains.^12^ Its unique target specificity, superior potency, and demonstrated efficacy in infection models make amycomicin an attractive lead for MDR-*S. aureus* therapy.^12^ Notably, amycomicin biosynthesis was first observed in a co-culture system in which *Amycolatopsis* sp. AA4 and *Streptomyces coelicolor* M145 interact metabolically; glucose conversion to galactose by the latter was reported to trigger amycomicin production. This context-dependent induction suggests that expression of the BGC may be tightly regulated and influenced by environmental or metabolic cues. Nevertheless, the amycomicin BGC was originally identified in *Amycolatopsis* sp. AA4 and has not been widely reported in *Streptomyces*, limiting the availability of established production hosts.^12^^,^^14^ *Streptomyces virginiae* (*S. virginiae*) has long been recognized for its metabolic versatility and the production of industrially relevant molecules.^15^ Yet its full genomic potential for synthesizing novel antibiotics remains insufficiently explored.

Here, we describe the isolation and multi-omics characterization of *S. virginiae* THA-960, a soil-derived strain exhibiting strong anti-MDR-*S. aureus* activity and harboring genomic signatures indicative of amycomicin biosynthesis. Through an integrated workflow combining phenotypic screening, complete genome sequencing, comparative phylogenomics across *Streptomyces* genomes, and metabolomic validation, we identify THA-960 as a putative amycomicin-producing strain with potent activity against clinical MDR-*S. aureus* isolates. This study provides a genome-guided antibiotic discovery framework that effectively links biosynthetic potential with functional antimicrobial output. By situating THA-960 within the broader *Streptomyces* phylogeny and assessing the taxonomic distribution of key BGCs, we introduce both a promising anti-MDR-*S. aureus* candidate and a systematic approach to accelerate natural product discovery in the continuing effort to combat antimicrobial resistance.

## Results

### Phenotypic screening reveals potent antimicrobial activity against MDR-*S. aureus*

**Figure 1:**
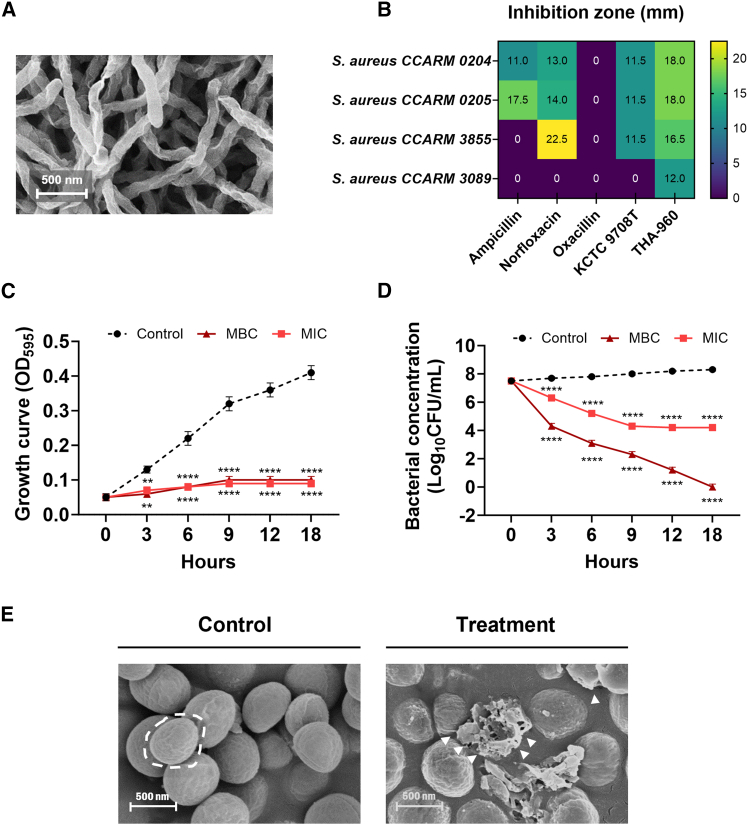
Antibacterial activity of the filtrate of *Streptomyces virginiae* THA-960 against MDR-*S. aureus* and multidrug-resistant strains (A) Scanning electron microscopy (SEM) image showing the morphology of *S. cinnamonensis* THA-960 (magnification: 20,000×; scale bars, 500 nm). (B) Inhibition zones (mm) in a disc diffusion assay of culture filtrate of *S. virginiae* THA-960 (and KCTC 9708^T^ reference strain) at 5 mg/disc compared with standard antibiotics (ampicillin (10 μg), norfloxacin (10 μg), and oxacillin (30 μg) per disc) against various multidrug-resistant *S. aureus* strains (CCARM 0204, 0205, 3855, 3089). All antibiotic concentrations are based on MIC values reported by the culture collection of antimicrobial resistant microbes (CCARM). (C) Growth curve of MRSA CCARM 3089 treated with culture filtrate of *S. virginiae* THA-960 at 15 mg/mL (MIC) and 30 mg/mL (MBC), indicating significant inhibition of bacterial growth compared with control. (D) Time-kill assay of MRSA CCARM 3089 exposed to 15 and 30 mg/mL of culture filtrate of *S. virginiae* THA-960, showing rapid and dose-dependent reduction in viable bacterial counts over 18 h. (E) Representative SEM images of MRSA CCARM 3089 cells: untreated control (left) and after treatment with culture filtrate of *S. virginiae* THA-960 (right). Treated cells show severe surface disruption and cell lysis (scale bars, 500 nm). MRSA in the control group appears as smooth, spherical cocci (dashed circle). THA-960 treatment induces surface blebbing and cell lysis (white arrows). Morphological changes are evident compared to the untreated control. Data are shown as mean ± SD. Statistical significance was analyzed by two-way ANOVA followed by Dunnett’s multiple-comparisons test comparing MIC- and MBC-treated groups with the untreated control at each corresponding time point; ∗∗*p* < 0.01 and ∗∗∗∗*p* < 0.0001 versus control (*n = 3*).

The culture filtrate of *S. virginiae* THA-960 demonstrated pronounced antimicrobial activity against a diverse panel of 17 MDR bacterial strains, notably including multiple MDR-*S. aureus* isolates (Table S1). All tested strains were resistant to at least three antibiotics, and THA-960 culture filtrate produced measurable inhibition zones against the MDR-*S. aureus* isolates examined in Figure 1.

In contrast, the THA-960 culture filtrate produced a clear 12 mm inhibition zone against culture collection of antimicrobial resistant microbes (CCARM) CCARM 3089, demonstrating unique efficacy where conventional approaches failed. For CCARM 0204 and CCARM 0205, THA-960 filtrate yielded the inhibition zones (18 mm each), exceeding all tested antibiotics and the positive *Streptomyces* control.

THA-960 filtrate was confirmed to have potent activity, with the lowest MIC of 0.08 mg/mL for CCARM 0204, whose minimum bactericidal concentration (MBC) was at 0.16 mg/mL, indicating bactericidal potency, while CCARM 0205 and CCARM 3855 had MICs of 0.16 mg/mL with MBCs of 0.31 mg/mL, and CCARM 3089 was less susceptible with MIC at 15 mg/mL and MBC at 30 mg/mL, correlating with the smallest inhibition zone (Table 1).

**Table 1:** MIC and MBC of *S. virginiae* THA-960 filtrate against *S. aureus* strains

Indicators	MIC (mg/mL)	MBC (mg/mL)
*S. aureus* CCARM 0204	0.08	0.16
*S. aureus* CCARM 0205	0.16	0.31
*S. aureus* CCARM 3855	0.16	0.31
*S. aureus* CCARM 3089	15	30

Time-kill kinetics further demonstrated bactericidal activity against CCARM 3089, with more than 3 log_10_ reduction in viable cell count observed within 18 h at both MIC and MBC concentrations, whereas the control maintained stable bacterial counts (Figure 1). Time-kill kinetics was measured up to 18 h, as >3-log reduction was achieved by 12 h with no regrowth. Scanning electron microscopy (SEM) revealed severe cell wall and membrane disruption in CCARM 3089 treated with THA-960, with treated cells collapsed, showing gross wall rupture and extensive debris, in contrast to smooth, intact cocci in untreated controls, confirming direct bactericidal action (Figure 1).

### Genomic mining predicts a rich repertoire of BGCs

**Figure 2:**
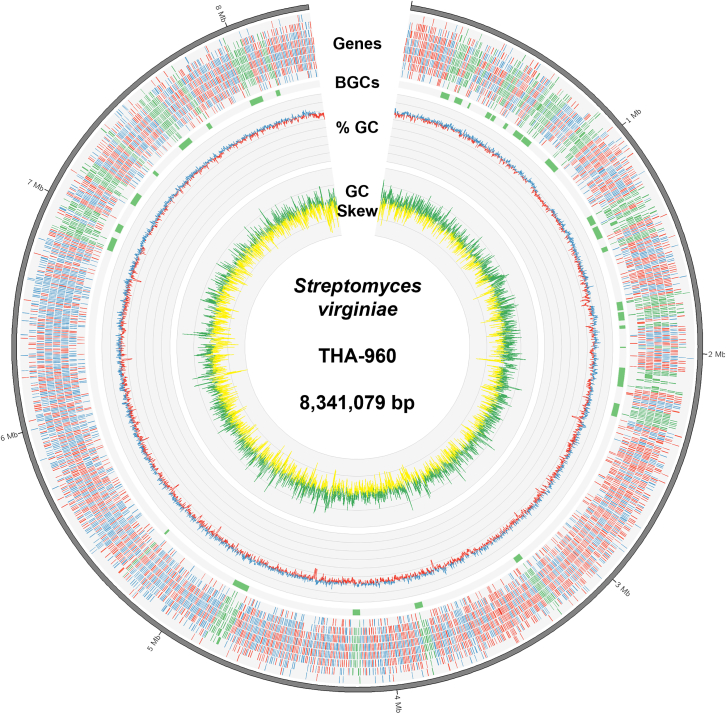
Genomic features of *Streptomyces cinnamonensis* strain THA-960 The diagram represents the chromosome of the strain THA-960 and is organized from the outermost track to the center as follows: (i) Predicted genes were represented by blue and red segments indicating the forward and reverse strands, respectively, with green segments indicating genes located within predicted biosynthetic gene clusters (BGCs) (predicted using antiSMASH v.8.0.4). (ii) A total of 31 predicted BGCs were indicated as green blocks, highlighting clusters potentially responsible for antibacterial and antitumor activities. (iii) GC content is displayed, with blue segments indicating regions above the average and red segments representing regions below the average. (iv) GC skew is shown, with green segments indicating positive values (>0) and yellow segments indicating negative values (<0), which can reflect replication origin and terminus regions.

The genome sequence was assembled in a single complete chromosome of 8,341,079 bp, exhibiting a guanine-cytosine (GC) content of 72.37%, a measure of DNA composition. Gene prediction identified 7,410 protein-coding genes, along with 91 tRNA and 21 rRNA genes within the genome sequence, which are essential for protein synthesis and ribosome function (Figure 2, Tables 2 and S2, and Data S1). A total of 7,195 predicted proteins were categorized into 22 clusters of orthologous groups (COGs). Around half (48.04%) of the total hits were designated as unknown function, meaning their roles in the cell are not yet determined. The other half of the hits were dispersed among diverse functional categories, with the highest counts in genes involved in transcription (543), amino acid transport and metabolism (390), signal transduction mechanisms (347), energy production and conversion (316), and carbohydrate transport and metabolism (316).

**Table 2:** Genomic features of strain THA-960 and *Streptomyces virginiae* NRRL B-8091

Genomic feature	1^a^	2^a^
Size of the genome assembly (bp)	8,341,079	9,185,056
GC content (%)	72.37	71.71
Protein-coding genes	7,410	8,516
CDS regions (bp)	7,358,160	8,008,985
Avg CDS (aa)	330	318
tRNA/rRNA genes	91/21	72/4
Complete BUSCOs (%)	100 (356/356)	99.7 (355/356)

a 1: THA-960, 2: *Streptomyces virginiae* NRRL B-8091 (GenBank:: GCF_000716685.1).

**Figure 3:**
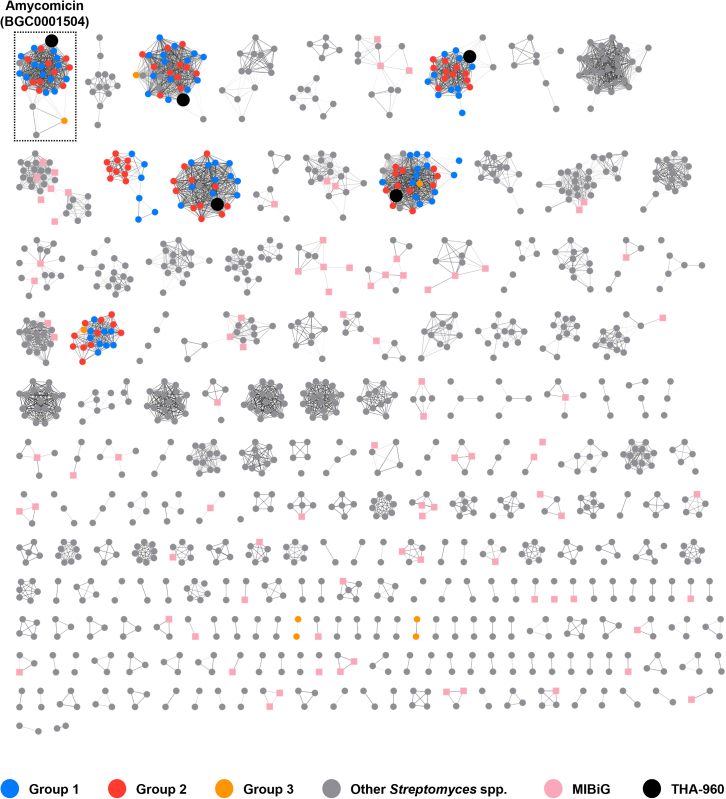
Sequence similarity network of PKS type I clusters generated using BiG-SCAPE The network encompasses 1,259 BGCs derived from the analysis of 521 selected *Streptomyces* genomes and MIBiG database. Nodes in the network are color-coded based on their taxonomic groups, which are shown at the bottom of the figure. The edges connecting the nodes are represented by a color gradient, reflecting the raw distances obtained from the BiG-SCAPE analysis. A component containing the predicted amycomicin cluster from strain THA-960 was indicated by dashed boxes. Dashed boxes highlight components containing predicted amycomicin and related clusters, suggesting evolutionary and functional conservation.

Furthermore, 196 hits fell into secondary metabolites biosynthesis, transport, and catabolism, suggesting the genomic potential for natural product biosynthesis (Table S3). The genomic potential for secondary metabolism was further affirmed through in silico identification of BGCs. A total of 31 BGCs were predicted from the genome sequence of strain THA-960 (Table S4). There were 10 candidate clusters for non-ribosomal peptide synthetase (NRPS), 10 for polyketide synthase (PKS), and seven for terpene products, respectively, often involved in defense or signaling. In addition, BGCs for several antibiotics, including amycomicin, streptothricin, and tambromycin, was identified from the genome sequence (Figure 3 and Table S4).

**Figure 4:**
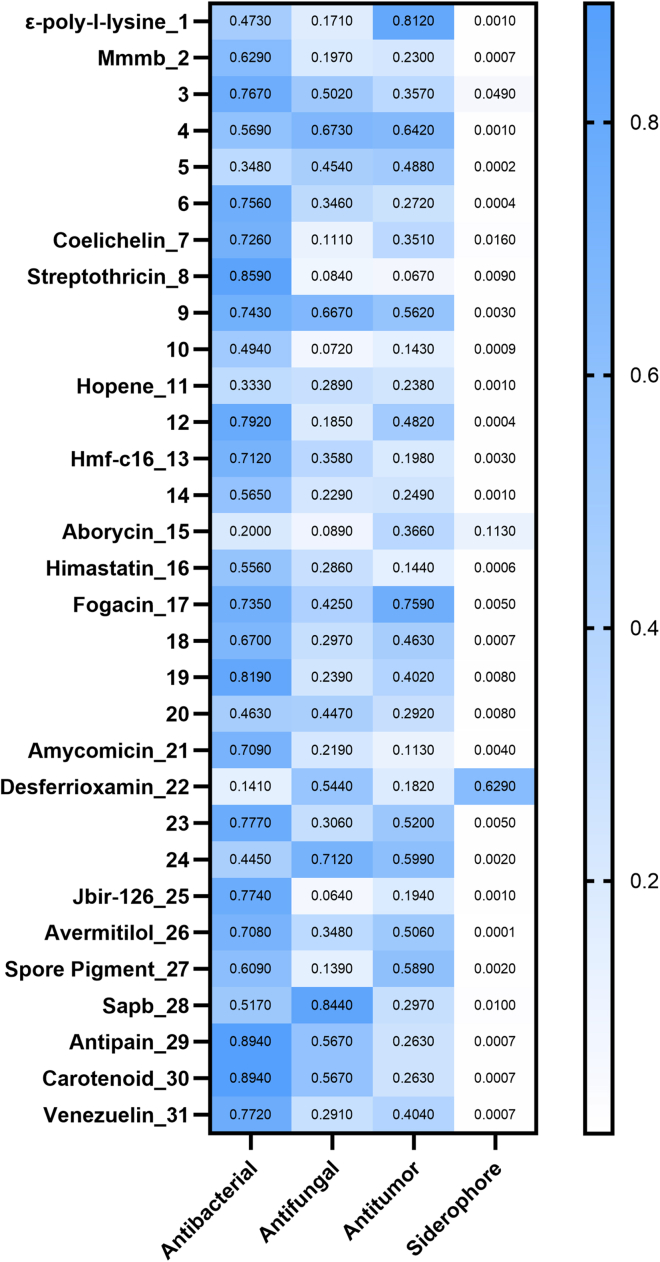
Heatmap of predicted bioactivity probabilities for biosynthetic gene clusters (BGCs) in *Streptomyces virginiae* THA-960 generated using NBPDetect Rows represent individual BGCs labeled by their most similar known cluster and cluster region numbers. Columns indicate predicted probabilities for antibacterial, antifungal, antitumor, and siderophore activities. Values ≥0.5 are considered positive predictions, and clusters with high dual activity are highlighted for prioritization. Probability scores range from 0 (low) to 1 (high), visualized by blue shading with darker tones indicating higher confidence. The heatmap highlights the diverse bioactivity potential within the THA-960 genome, including clusters with strong predicted antibacterial and antitumor activity that may warrant further investigation. This analysis integrates genome-based predictions with known BGC functions to identify potential bioactive compounds.

Bioactivity prediction using NBPDetect identified several BGCs in *S. virginiae* THA-960 with notable predicted probabilities for antibacterial and antitumor activities (Figure 4). Among these, Amycomicin_21 exhibited a balanced profile, with an antibacterial probability of 0.709 (70.9%, approximately 1.5-fold higher than the cluster average of 0.46) and an antitumor probability of 0.413 (41.3%, approximately 1.15-fold higher than the mean of 0.36), making it one of the clusters with above-average predictions in both categories. While Streptothricin_8 and Antipain_29 showed higher antibacterial probabilities (0.865 [86.5%] and 0.894 [89.4%], respectively; approximately 1.9-fold higher than the mean), their antitumor probabilities were lower (56.2% and 26%, respectively). Other clusters, such as Sapb_28 (antibacterial 0.517, 51.7%; dual activity exceeding 26%) and Jbir-126_25 (antibacterial 0.774, 77.4%), contributed to the overall antibacterial potential but displayed less balanced dual activity.

On the antitumor axis, Fogacin_17 reached the highest probability (0.779, 77.9%; more than 2-fold higher than the mean), whereas its antibacterial probability (0.35, 35%) was lower than that of Amycomicin_21. This comparison highlights Amycomicin_21 as a cluster with moderate to high probabilities in both antibacterial and antitumor activities, rather than extreme specialization in a single activity. The siderophore cluster Desferrioxamin_22 showed a high siderophore-specific probability of 0.629 (62.9%), markedly above the average of approximately 0.02, but it was functionally distinct from the antibacterial and antitumor clusters. Overall, these predictions indicate that Amycomicin_21 represents a well-balanced dual-activity cluster in THA-960, alongside other dual-activity clusters, supporting its prioritization for further functional characterization (Figure 4).

### Phylogenomic analysis for species identification

**Figure 5:**
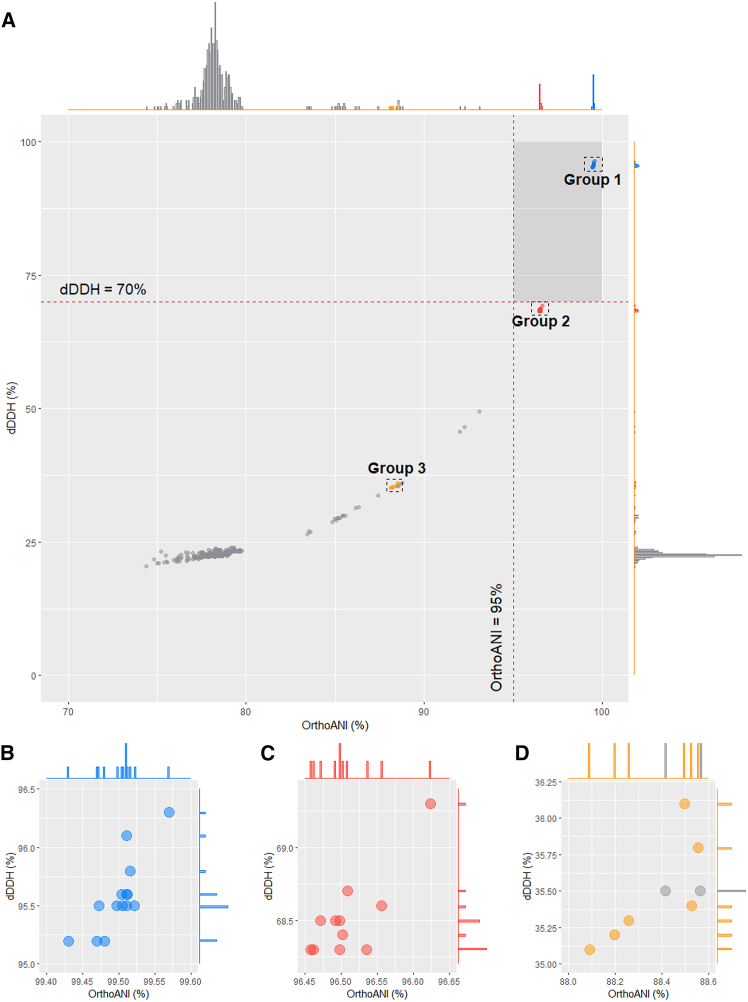
Distribution of genomic relatedness for strain THA-960 and the selected *Streptomyces* spp (A) The scatterplot illustrates the distribution of OrthoANI and dDDH values calculated for strain THA-960 and 520 *Streptomyces* spp. The *x* and *y* axes represent the calculations of OrthoANI and dDDH against the genome sequence of strain THA-960, respectively. Magnified views of the dashed boxes, group 1 (B), group 2 (C), and group 3 (D). These analyses provide context for taxonomic placement and genomic diversity within *S. virginiae.* Summary of genome counts following quality filtering. The final dataset included 489 *Streptomyces* genomes (chromosome-level or high-quality assemblies), 10 genomes classified as *S. virginiae* according to NCBI taxonomy, 11 genomes highly similar to strain THA-960 (sensu lato), and 10 genomes highly similar to the NCBI reference genome (sensu stricto), including the reference itself.

The 16S rRNA gene sequences were analyzed to identify strain THA-960. The consensus sequence derived from the seven 16S rRNA genes predicted in the genome exhibited sequence identities exceeding 99.7% when compared to those of *S. virginiae* NRRL ISP-5094, *Streptomyces xanthophaeus* NRRL B-5414, *Streptomyces nojiriensis* LMG 20094, and *Streptomyces spororaveus* LMG 20313 (Table S2). In the phylogenetic tree constructed from the 16S rDNA sequences, strain THA-960 was positioned as a sister taxon to *S. virginiae* NRRL ISP-5094, while *S. manipurensis* Microbial Biotechnology Research Laboratory (MBRL) 201 occupied a basal position (Figure S1). Although a sequence identity cutoff of 97%–98% for 16S rRNA gene sequences has been commonly employed in species identification,^16^^,^^17^ supplementary analyses have demonstrated that bacterial strains sharing high sequence similarity might still be classified as different species.^18^^,^^19^^,^^20^ Hence, genomic similarity indices, including dDDH and OrthoANI, were computed for a set of 520 *Streptomyces* genome sequences (Data S1). Among the genomes, 14 exhibited OrthoANI values exceeding 99% (group 1; Figure 5B and Data S1), implying the possibility that they belong to the same species as strain THA-960. Out of the 14 genomes, three belong to *S. virginiae*. However, one was designated as *S. lavendulae* subsp. *lavendulae*, and the remaining ten were classified as unassigned strains (Data S1). Moreover, eleven closely related genomes exhibited OrthoANI values ranging between 96% and 97% (group 2; Figure 5C and Data S1), although they slightly fell below the species demarcation threshold for dDDH (Data S1).

Surprisingly, six of the ten genomes designated as *S. virginiae* in NCBI RefSeq database were not part of these two groups, displaying OrthoANI values less than 90% when compared to the THA-960 genome (group 3; Figure 5D and Data S1). Pairwise OrthoANI calculations among the six genomes showed a wide spectrum of genomic identity (Figure S2), implying that they may form distinct species different from the aforementioned group 1 and 2.

### Distribution of BGCs across the 521 *Streptomyces* genomes

A comprehensive analysis was carried out on 41,002 protein sequences linked to 2,502 BGCs, comparing them against 521 genome sequences (Data S1). Notably, the biosynthetic genes for virginiamycin S1 (BGC0001116) were uniquely identified in the genome of *S. virginiae* NRRL B-8091 (GenBank: GCF_000716685.1), indicating that this compound is specific to a particular strain rather than being common in *S. virginiae* species (Figure S3). In contrast, the BGCs for certain antibiotics showed a varied presence across *S. virginiae* groups 1–3, suggesting they could be key metabolic markers for these taxa (Figures 5, S4, and S5). The streptothricin gene cluster (BGC0000432) was consistently present in several species, including group 1 of *S. virginiae*, but absent in groups 2 and 3 (Figure 5A).

Tambromycin cluster (BGC0001368) exhibited a strong conserveness across groups 1–3, yet it was surprisingly absent in other Streptomyces genomes used in the study (Figure 4B). Four genomes from group 3 (GenBank: GCF_026340475.1, GCF_026341715.1, GCF_026342285.1, and GCF_026342475.1) lacked the gene for 4′-phosphopantetheinyl transferase (PPTase; accession: WP_051700107.1). Interestingly, these group 3 strains were genetically similar to *S. nojiriensis* Japan Collection of Microorganisms (JCM) 3382 (Figure S6), known for producing tambromycin.^21^ Lastly, amycomicin cluster (BGC0001504) was found in genomes from groups 1 and 2, but not in group 3 (Figure 5C).

### Network analysis on BGCs and their co-occurrence

**Figure 6:**
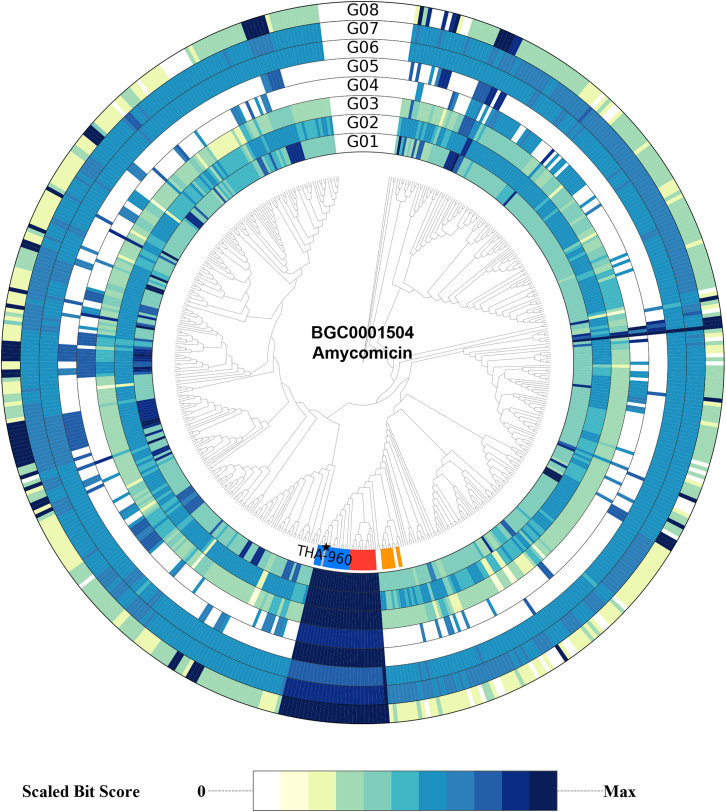
Distribution of the amycomicin biosynthetic gene cluster across *Streptomyces virginiae* genomes Protein sequences from the amycomicin cluster were searched using TBLASTN (E-value < 1e−5) against the genomes of strain THA-960 and selected *Streptomyces* strains. Dark navy-blue shading indicates the maximum bit score for each protein, reflecting the degree of sequence homology, with darker colors representing stronger conservation. Strain THA-960 is highlighted with a filled-star mark at the terminal node. This shows which strains may produce similar bioactive compounds.

A total of 16,484 BGCs were identified from the genomic data of strain THA-960 and 520 other *Streptomyces* strains (Data S1). While the genomes within groups 1–3 exhibited distinct genomic characteristics, the analysis of BGCs revealed a close relationship among them. This phenomenon was particularly evident in the network analysis of NRPS and PKS clusters, which have been well-known for their involvement in antibiotic biosynthesis.^1^ The BGCs were organized into multiple components, primarily comprising clusters from genomes within groups 1–3. For instance, streptothricin clusters formed a component that included species from groups 1–2 and a few other strains. Similarly, tambromycin clusters were predominantly composed of BGCs from species within groups 1–3 (Figure S7). This trend was also observed in the sequence similarity network of PKS type I clusters derived from the 521 *Streptomyces* genomes. An example of this pattern can be seen in a component that encompassed amycomicin clusters, primarily consisting of BGCs from species in groups 1–3 (Figures 6 and S8).

**Figure 7:**
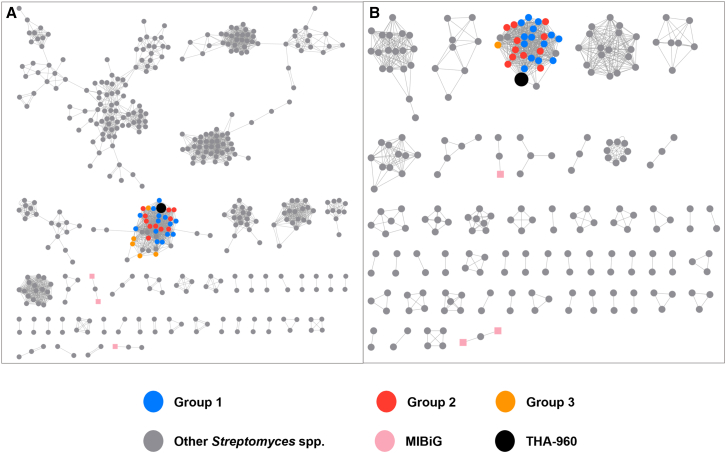
Co-occurrence networks of *Streptomyces* genomes, specifically highlighting NRPS and PKS type I gene clusters These networks offer visual insights into the extent of co-occurrence observed in the sequence similarity networks of NRPS (A) and PKS type I clusters (B). The degree of co-occurrence was quantified by counting the number of homologous gene clusters between two genomes, assessed pairwise from the sequence similarity networks. Nodes represent genomes; edges represent the number of shared homologous BGCs. Higher connectivity indicates closer functional similarity in secondary metabolite potential.

Interestingly, BGCs from groups 1–3 frequently co-occurred, setting them apart from other streptomyces species. To gain deeper insights into the biosynthetic relationships among genomes in groups 1–3, a co-occurrence network was constructed. In this network, nodes represented genomes, and edges represented the number of homologous gene clusters between two genomes based on sequence similarity networks (Figures 5, S7, and S8). All genomes within groups 1–3, with the exception of one from group 3 (GenBank: GCF_000716685.1), formed a component along with a few other *Streptomyces* genomes. Moreover, all 14 genomes from group 1 and 11 from group 2 clustered together in a component within the co-occurrence network of PKS type I clusters. Notably, the genome sequence of *S. virginiae* NRRL B-1447 (GenBank: GCF_001270565.1) was the only one from group 3 present in this component (Figure 7). These findings suggest that groups 1 and 2 share a high degree of similarity in their biosynthetic machinery despite differences in their genomic makeup. In contrast, group 3 exhibits less homology in terms of biosynthetic potential with groups 1 and 2, consistent with the results obtained from phylogenomic analysis.

### Putative antimicrobial-related metabolites detected by LC-QTOF-MS

**Figure 8:**
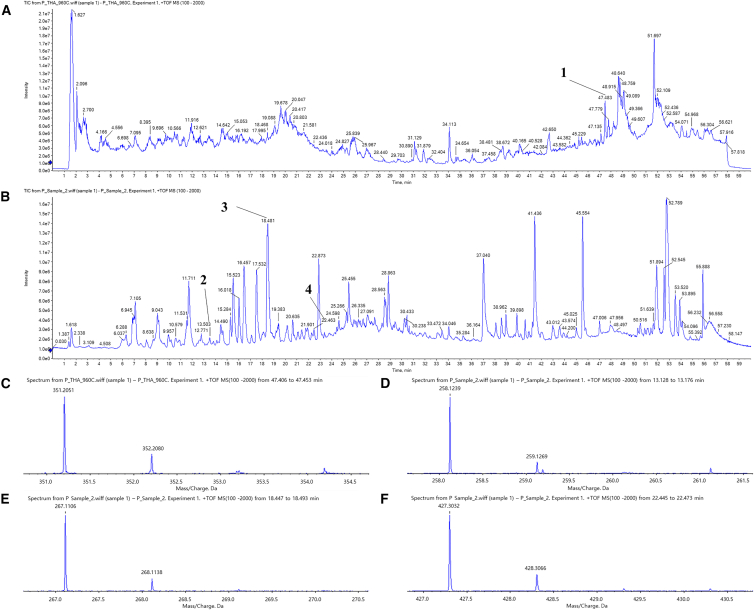
ESI-positive TIC and mass spectrum of *Streptomyces virginiae* THA-960 Total ion chromatogram of cell (A), and broth (B) extract of *S. virginiae* THA-960; mass spectrum of amycomicin (C), indolmycin (D), tubercidin (E), and leupeptin (F) labeled with 1–4.

To analyze the metabolites of strain THA-960, cell and culture broth extracts were separately prepared and analyzed using UPLC-Qtof-MS. Liquid chromatography–quadrupole time-of-flight mass spectrometry (LC-QTOF-MS) analysis detected a metabolite feature putatively assigned as amycomicin in the cell extract and indolmycin, tubercidin, leupeptin in the culture broth extract (Figure 8 and Table 3). Based on the analysis of mass spectrometry (MS) data, amycomicin was putatively identified through accurate mass matching and metabolomic profiling, consistent with one of the three antimicrobial compounds predicted by genome analysis. This substance was detected in electrospray ionization (ESI)-positive mode at repetition time (RT) 47.483 min, and the MS spectrum is depicted in Figure 8. The observed m/z was consistent with the expected exact mass of amycomicin; however, this assignment remains preliminary in the absence of additional structural validation. Therefore, the genomic evidence in the present study specifically supports the amycomicin candidate, whereas the other filtrate metabolites are presented as tentative LC-QTOF-MS assignments only.

**Table 3:** Identified antimicrobial components of *S. virginiae* THA-960

Category	No.	Components	RT (min)	Formula	Expected *m/z*	Observed *m/z*	Mass error (mDa)
Cell	1	Amycomicin	47.43	C_19_H_29_NO_5_	351.2046	351.2051	1.4
Filtrate	2	Indolmycin	13.15	C_14_H_15_N_3_O_2_	258.1237	258.1239	0.2
	3	Tubercidin	18.47	C_11_H_14_N_4_O_4_	267.1088	267.1106	1.82
	4	Leupeptin	22.47	C_20_H_38_N_6_O_4_	427.3027	427.3032	0.47

### AI-based structural modeling identifies high-confidence amycomicin targets in MDR-*S. aureus*

To explore the structural compatibility of amycomicin with FabH and selected Methicillin-resistant *Staphylococcus aureus* (MRSA)-associated proteins, protein-ligand complex models were generated using AlphaFold3 (AF3), and model confidence metrics were evaluated (Table S6). Structural reliability was assessed using predicted template modeling score (pTM), interface pTM (ipTM), predicted local distance difference test (pLDDT), and predicted aligned error (PAE).

Because amycomicin was previously demonstrated to selectively inhibit the β-ketoacyl-acyl carrier protein synthase III FabH in *S. aureus*,^12^ FabH was included as a validated reference target. In addition, representative essential MRSA proteins spanning major antibacterial target classes were examined in an exploratory manner, including DNA replication (DNA gyrase subunit A), transcription (RNA polymerase β subunit), and translation (elongation factor Tu), which are well-established antibiotic target categories^22^ and frequently implicated in resistance development in *S. aureus*.^23^

FabH (UniProt: Q6GAU3), encoded by *fabH*, exhibited the highest structural confidence (pTM = 0.95; ipTM = 0.91), with global pLDDT >95 and interface pLDDT >81. Interface PAE values were low (approximately 3Å), supporting precise ligand positioning. Interaction profiling identified 5 hydrogen bonds, 8 hydrophobic interactions, and 14 additional contacts, with a predicted binding free energy of −9.6 kcal/mol, consistent with its experimentally validated inhibition.

DNA gyrase subunit A (GyrA; UniProt: P0A0H3), encoded by *gyrA*, demonstrated high-confidence metrics (pTM = 0.6; ipTM = 0.82) and favorable predicted binding energy (*ΔG* = −7.05 kcal/mol). Similarly, RNA polymerase β subunit (RpoB; UniProt: P00807; *ΔG* = −8.61 kcal/mol) and Cfr methyltransferase (cfr; UniProt: A5HBL2; *ΔG* = −9.47 kcal/mol) displayed strong interface confidence (ipTM ≥0.87) with low PAE values, supporting stable ligand engagement.

## Discussion

This study establishes *S. virginiae* THA-960 as both a potent anti-MDR-*S. aureus* resource and a paradigm for integrated genome-guided antibiotic discovery. By coupling the first complete genome of *S. virginiae* with comprehensive metabolomic profiling and phylogenomic analysis across 520 *Streptomyces* strains, we demonstrate that THA-960 represents a potent antibiotic producer with activity against clinical MDR-*S. aureus* isolates. Metabologenomic analysis revealed the genomic and metabolomic repertoire of antimicrobial activity of strain THA-960.

Globally, the indiscriminate use of antibiotics has led to an escalation in drug resistance, thereby amplifying the demand for new antimicrobial agents. The diversification of antibiotic-resistant strains underscores the imperative for the discovery of novel antimicrobial compounds. Our study substantiated a broad spectrum of antimicrobial activity against 17 MDR bacterial strains (Table S1). Moreover, exceptional efficacy against prevalent MDR-*S. aureus* was established, and scanning electron microscopy analysis revealed the destruction of MDR-*S. aureus* (Figure 1).

Previous studies suggest that Methicillin-susceptible *Staphylococcus aureus* (MSSA) or MRSA classification alone does not always explain how a strain responds to a new antimicrobial agent. The standard MSSA strain American Type Culture Collection (ATCC) 25923 and the standard MRSA strain ATCC 43300 have been reported to show similar susceptibility to some newly tested compounds.^24^ By contrast, clinical isolates such as the CCARM strains often show much greater variation, with some strains responding well and others showing much weaker susceptibility.^25^ Consistent with this pattern, our own CCARM panel also showed broad variation in response to THA-960, ranging from 0.08 to 0.16 mg/mL for CCARM 0204 to 15 to 30 mg/mL for CCARM 3089, with CCARM 0205 and CCARM 3855 showing intermediate susceptibility (Table 1).

Previous studies indicate that amycomicin production requires interspecies interaction and metabolic activation during co-culture. In contrast, THA-960 produced a metabolite with a molecular mass consistent with amycomicin under monoculture conditions, suggesting that this strain harbors regulatory or metabolic features that enable expression of the amycomicin BGC without interspecies induction. Putative amycomicin production by THA-960 represents a significant discovery, particularly given its documented nanomolar potency against MDR-*S. aureus*, more than 100-fold stronger than most known actinomycete-derived antibiotics.^12^ While previous culture filtrates with anti-MDR-*S. aureus* typically present MICs in the 0.1–2 mg/mL range,^26^^,^^27^ the confirmed production of amycomicin by THA-960 provides a mechanistic explanation for the observed superior activity. Our integrated approach, detecting both the complete BGC and the actual metabolite, supports genome-guided discovery compared to traditional bioassay workflows and explains why THA-960 outperforms related strains like *S. cinnamonensis* KCTC 9708^T^ by over 43.5%.

It was reported that *S. cinnamonensis* and *S. virginiae* were different names referring to the same species identified from multiple sources.^2^ Therefore, our study aimed to validate the efficacy by comparing the antimicrobial activity of *S. virginiae* THA-960 with the type strain of *S. cinnamonensis*. The results demonstrated that *S. virginiae* THA-960 exhibited over 43.5% superior activity at the same concentrations (Table 1). Based on these findings, *S. virginiae* THA-960 was postulated to produce distinct antimicrobial compounds compared to the sequenced *S. virginiae*. Metabolite profiling results through UPLC-Qtof-MS revealed the presence of compounds distinct from those previously known to be produced by *S. virginiae*, such as virginiamycin and monensin; instead, amycomicin, indolmycin, tubercidin, and leupeptin were identified (Table 2). The antibacterial activity observed in the crude culture filtrate may therefore reflect the combined effects of multiple bioactive metabolites produced by THA-960. While metabolomic analysis detected a compound with an exact mass consistent with amycomicin, definitive structural confirmation requires additional analytical validation such as MS/MS fragmentation analysis, purification, and NMR characterization. Particularly noteworthy was amycomicin, despite not being currently characterized or commercially available, which was confirmed through genetic analysis to possess the BGC related to amycomicin production in *S. virginiae* THA-960. Therefore, optimization of culture conditions may facilitate the isolation and purification of substantial quantities of high-purity amycomicin. Zheng et al. elucidated amycomicin biosynthesis, showing that the pathway involves AmcQ, AmcB, and triple P450s (AmcC/D/B) to form isonitrile, epoxy, and keto motifs, providing mechanistic insight into the chemical structure predicted from the THA-960 BGC.^14^

Beyond biosynthetic confirmation, structural modeling extended our genome-guided discovery framework to a limited mechanistic level. Amycomicin was previously identified as a selective inhibitor of β-ketoacyl-acyl carrier protein synthase III (FabH), a key enzyme initiating the type II fatty acid biosynthesis (FASII) pathway in *S. aureus*. This FabH-centered mechanism is further supported by Pishchany et al.,^12^ who showed that introducing *Bacillus subtilis* fabHB into *S. aureus* markedly increased resistance to amycomicin, whereas fabHA or a catalytically inactive fabHB mutant did not confer comparable resistance. Consistent with this experimentally validated mechanism, AF3-based protein-ligand complex prediction demonstrated high-confidence interaction between amycomicin and FabH, as reflected by elevated pTM/ipTM values, strong interface pLDDT scores, low PAE, and favorable binding free energy. To contextualize its possible protein-level structural compatibility in MDR-*S. aureus*, selected non-membrane-associated essential proteins representing major antibacterial target classes were examined exploratorily, including DNA gyrase subunit A (GyrA), RNA polymerase β subunit (RpoB), and elongation factor Tu (EF-Tu), which are canonical targets of fluoroquinolones, rifamycins, and translation inhibitors, respectively. AF3 predictions indicated structurally plausible interactions with these proteins, but these interactions remain computational predictions and require experimental validation. Thus, amycomicin is best interpreted as acting through its previously validated FabH-related mechanism, while any additional cellular interactions should be considered hypothetical and likely limited *in vivo* unless supported by future validation using purified compounds and genetically characterized MDR-*S. aureus* strains. Such putative multi-target structural compatibility may contribute to the pronounced activity observed against clinical MDR-*S. aureus* isolates and could impose a higher evolutionary barrier to resistance development.

Phylogenomic analysis suggested that *S. virginiae* might encompass multiple taxonomic groups with a wide range of genomic similarity (Figure 5). The majority of the ten genomes identified as *S. virginiae* in NCBI RefSeq database were equivocally identified with low genomic identity to the type strains (Table S5). It was also demonstrated by this study, suggesting that there were three taxonomic groups based on genomic similarity indices (Figure 5 and Data S1). These genetically diverse taxonomic groups might render functional diversification in biosynthetic potential of natural products. Chen et al. positioned THA-960 in a clade of plant-beneficial *Streptomyces*, confirming its identity as *S. cinnamonensis* and suggesting potential ecological and biocontrol roles beyond anti-*S. aureus* activity.^28^

Dynamic conserveness distribution of BGCs for amycomicin, streptothricin, and tambromycin demonstrated functional diversity in *S. virginiae*. Strains in groups 1–3 were more closely related in terms of biosynthetic signatures than genomic identities; however, they showed subtle differences in the distribution of antibiotic clusters (Figure 6). Interestingly, a gene encoding putative PPTase was missing in the four genomes of group 3 (Figure 5B). A gene, *sfp*, encoding a PPTase in *Bacillus subtilis* was known to be involved in the production of non-ribosomal peptides and polyketide biosynthesis.^3^ The presence and absence of this gene may be an example of the biosynthetic diversification occurring in genome sequences. Meanwhile, more BGCs were showing a high level of conserveness across groups 1–3 (Figure S3), for example, gene clusters of fusaricidin B (BGC0001152) and fogacins (BGC0002021). This suggested that taxonomic groups proposed in this study, groups 1–3, may hold ample biosynthetic potential, thus exhibiting strong antimicrobial activities. Future studies involving targeted gene disruption, transcriptional analysis, or heterologous expression of the BGC will be necessary to conclusively verify amycomicin biosynthesis and its contribution to antimicrobial activity.

### Limitations of the study

In-depth comparative phylogenomic analysis unveiled the intricate taxonomy of *S. virginiae,* suggesting the presence of multiple distinct groups. Notably, one of these groups exhibits >99% OrthoANI similarity when compared to the genome sequence of strain THA-960. The efficacy of strain THA-960 against MDR bacteria, including an MDR-*S. aureus* strain was unequivocally demonstrated through multiple bioassays. Metabologenomic analysis provided a comprehensive insight into potential compounds and their underlying genetic machineries. Specifically, amycomicin was confidently identified through genome mining and metabolomic analysis showed a peak that might correspond to amycomicin. These findings underscore that THA-960 exhibits antimicrobial activity primarily through the production of antibiotic small molecules, showcasing its potential as a valuable source for combating a broad spectrum of drug-resistant bacteria, especially MDR-*S. aureus* strains. In the absence of purified standard comparison and MS/MS-based structural confirmation, additional analytical and genetic validation will be necessary to confirm amycomicin production conclusively. This condition likely reflects the current limits of genome-mining annotation and database-based cluster recognition. In addition, the identification of amycomicin was based on high-resolution MS and genome mining; further structural and genetic validation is required for definitive confirmation.

## Resource availability

### Lead contact

Further information and requests for resources and reagents should be directed to and will be fulfilled by the lead contact, Tae-Hoo Yi (drhoo@khu.ac.kr).

### Materials availability

All materials used in this study, including *S. virginiae* THA-960, are deposited in the laboratory collection of Professor Tae-Hoo Yi at Kyung Hee University, Republic of Korea. Materials are available from the lead contact upon reasonable request, subject to institutional and regulatory requirements.

### Data and code availability

• Genome sequence data for *S. virginiae* THA-960 have been deposited in NCBI GenBank and are publicly available as of the date of publication. The accession numbers are listed in the key resources table.
• This paper does not report original code.
• Any additional information required to reanalyze the data reported in this paper is available from the lead contact upon request.

## Acknowledgments

This work was supported by the 10.13039/501100003725National Research Foundation of Korea (NRF) grant funded by the Korean Government (10.13039/501100014188MSIT) (RS-2025-24482968).

## Author contributions

T.T.M.N., investigation, methodology, formal analysis, writing – original draft; J.J., investigation, writing – original draft; X.J. and Q.Z., investigation; J.C., investigation, methodology, formal analysis, supervision, writing – original draft, writing – review and editing; T.-H.Y., conceptualization, supervision, writing – review and editing; All authors have read and approved the final manuscript.

## Declaration of interests

The authors declare no competing interests.

## Declaration of generative AI and AI-assisted technologies in the writing process

During the preparation of this work, the authors used ChatGPT (OpenAI) to improve readability and language. After using this tool, the authors reviewed and edited the content as needed and take full responsibility for the content of the published article.

## STAR★Methods

### Key resources table

**Table:** 

REAGENT or RESOURCE	SOURCE	IDENTIFIER
**Bacterial and virus strains**		

*Streptomyces virginiae* THA-960	Laboratory collection of Tae-Hoo Yi, Kyung Hee University	THA-960
*Streptomyces cinnamonensis* KCTC 9708^T^	Korean Collection for Type Cultures	KCTC 9708^T^
*Enterococcus faecalis* CCARM 5171	Culture Collection of Antimicrobial Resistant Microbes	CCARM 5171
*Enterococcus faecium* CCARM 5262	Culture Collection of Antimicrobial Resistant Microbes	CCARM 5262
*Staphylococcus aureus* CCARM 0204	Culture Collection of Antimicrobial Resistant Microbes	CCARM 0204
*Staphylococcus aureus* CCARM 0205	Culture Collection of Antimicrobial Resistant Microbes	CCARM 0205
*Staphylococcus aureus* CCARM 3855	Culture Collection of Antimicrobial Resistant Microbes	CCARM 3855
*Staphylococcus aureus* CCARM 3089	Culture Collection of Antimicrobial Resistant Microbes	CCARM 3089
*Escherichia coli* DC 0 CCARM 0237	Culture Collection of Antimicrobial Resistant Microbes	CCARM 0237
*Escherichia coli* DC 2 CCARM 0238	Culture Collection of Antimicrobial Resistant Microbes	CCARM 0238
*Escherichia coli* TEM CCARM 0235	Culture Collection of Antimicrobial Resistant Microbes	CCARM 0235
*Escherichia coli* 1507 CCARM 0236	Culture Collection of Antimicrobial Resistant Microbes	CCARM 0236
*Pseudomonas aeruginosa* CCARM 0223	Culture Collection of Antimicrobial Resistant Microbes	CCARM 0223
*Pseudomonas aeruginosa* CCARM 0224	Culture Collection of Antimicrobial Resistant Microbes	CCARM 0224
*Salmonella enterica* subsp. enterica serovar Typhimurium CCARM 0240	Culture Collection of Antimicrobial Resistant Microbes	CCARM 0240
*Klebsiella oxytoca* CCARM 0248	Culture Collection of Antimicrobial Resistant Microbes	CCARM 0248
*Klebsiella aerogenes* 1522E CCARM 0249	Culture Collection of Antimicrobial Resistant Microbes	CCARM 0249
*Enterobacter cloacae* P99 CCARM 0252	Culture Collection of Antimicrobial Resistant Microbes	CCARM 0252
*Enterobacter cloacae* 1321E CCARM 0253	Culture Collection of Antimicrobial Resistant Microbes	CCARM 0253

**Chemicals, peptides, and recombinant proteins**		

Starch casein agar (SCA)	Kisanbio	Cat# MB-S0610
Starch casein broth (SCB)	Kisanbio	Cat# MB-S0730
Mueller–Hinton agar (MHA)	BD Difco	Cat# 225250
Glutaraldehyde	Daejung	Cat# 111-30-8
Osmium tetroxide 1%	Sigma-Aldrich	Cat# 20816-12-0
Ethyl alcohol	Daejung	Cat# 64-17-5
Hexamethyldisilazane (HMDS)	Sigma-Aldrich	Cat# 999-97-3
Ampicillin	MERCK	Cat# 69-53-4
Oxacillin	MERCK	Cat# 7240-38-2
Norfloxacin	MERCK	Cat# 70458-96-7

**Deposited data**		

THA-960 genome assembly	NCBI GenBank	GCA_036870975.1
BioProject of THA-960	NCBI BioProject	PRJNA1077906

**Software and algorithms**		

SMRT Link	PacBio	v11.1.0
Pilon	GitHub	v1.21
BUSCO	BUSCO	v5.4.7
Prokka	GitHub	v1.14.6
RNAmmer	Technical University of Denmark	v1.2
Circos	Circos	v0.69-9
eggNOG-mapper	GitHub	v2.1.13
antiSMASH	antiSMASH	v8.0.4
MEGA-CC	MEGA Software	v10.2.6
OrthoANI	GitHub	v1.40
GGDC	DSMZ	v3.0
R (ggplot2, ggExtra, ggthemes)	R Core Team	v4.2.3
CVTree	GitHub	CVTree3
GraPhlAn	GitHub	v1.1.4
BiG-SCAPE	GitHub	v2.0.0
Cytoscape v3.10.0	Cytoscape Consortium	v3.10.0
NPBDetect v1.0	GitHub	v1.0
GraphPad Prism	GraphPad Software, LLC	V10

### Experimental model and study participant details

This study used microbial strains as experimental models. No human participants, human samples, animals, plants, primary cell cultures, or immortalized cell lines were used in this study. The antibiotic-producing strain *Streptomyces virginiae* THA-960 was isolated from soil collected near a mudflat on the west coast of Buan-gun, Jeollabuk-do, Republic of Korea. Soil suspensions were serially diluted, spread onto starch casein agar (SCA), and incubated at 30°C for 3 days. A single Streptomyces colony was selected and cultured in starch casein broth (SCB) for subsequent experiments. For preparation of antimicrobial culture filtrate, *S. virginiae* THA-960 was cultured in SCB for 24 h as a seed culture. Then, 1% of the seed culture was inoculated into fresh SCB and cultured for 7 to 10 days at 30°C. The culture broth was centrifuged at 12,000 rpm for 20 min and filtered through a 0.22 μm membrane before use.

*Streptomyces cinnamonensis* KCTC 9708^T^ was used as a reference *Streptomyces* strain and was treated at the same concentration as *S. virginiae* THA-960. Multidrug-resistant strains were obtained from the Culture Collection of Antimicrobial Resistant Microbes (CCARM).

For disc diffusion assays, bacterial indicators were spread on Mueller–Hinton agar (MHA) at 1 × 10^6^ CFU/mL, treated with test samples or antibiotics, and incubated at 30°C for 24 h. For growth-curve and time-kill assays, MDR-S. aureus was incubated in nutrient broth at 37°C for 0–24 h. MIC and MBC assays were performed using the broth microdilution method according to CLSI guidelines with four *S. aureus* strains and incubated at 37°C for 24 h. For scanning electron microscopy analysis, *S. aureus* CCARM 3089 was treated with culture filtrate at the MIC concentration or left untreated as a control and incubated at 37°C for 12 h before fixation and imaging.

Genotype and age/developmental stage were not applicable to the bacterial strains used in this study. Biological sex and gender were also not applicable because bacteria do not have biological sex or gender as defined for human, animal, or eukaryotic cell-line models; therefore, the influence of sex or gender on the results could not be assessed. Because no human participants, human samples, animals, plants, primary cell cultures, or immortalized cell lines were used, institutional approval for human or animal subjects, subject/sample allocation, cell-line sex, cell-line authentication, and mycoplasma testing were not applicable.

The identity of *S. virginiae* THA-960 was assessed by 16S rRNA gene sequence analysis and genome-based taxonomic analyses, including OrthoANI and digital DNA–DNA hybridization. The MDR strains were used according to their CCARM strain identifiers. No additional cell-line authentication was performed because no eukaryotic cell lines were used.

### Method details

#### Isolation of a bacterial strain THA-960

Soil samples were collected from the vicinity of the mudflat on the west coast of Buan-gun, Jeollabuk-do, Republic of Korea. Each sample, quantified at 1 g, was dissolved in 9 mL of sterile water. Subsequently, the sample was subjected to dilution ranging from 10^-3^ to 10^-5^ using sterile water. A volume of 100 μL from each dilution was spread onto starch casein agar medium (Kisanbio Co., Ltd., Seoul, Republic of Korea) and then incubated at 30°C for 3 days. Following incubation, a single Streptomyces colony was isolated and cultured in starch casein broth (Kisanbio Co., Ltd., Seoul, Republic of Korea) for experimentation.

#### Antimicrobial activity on MDR bacteria

The isolated THA-960 was cultured in SCB medium for 24 h. After that, 1% of seed culture broth was main cultured in SCB medium more than 7 days. After 7 − 10 days, the medium was centrifuged at 12,000 rpm for 20 min, and filtered through 0.22 μm filter (Hyundai micro Co., Ltd., Anseong-si, Gyeonggi-do, Republic of Korea).

Disc diffusion was performed to confirm the antimicrobial activity against 17 bacterial strains including MDR bacteria (Table S1). Ampicillin, oxacillin, norfloxacin, and the *S. cinnamonensis* KCTC 9708^T^ was used as control group. The concentrations of the antibiotics were treated based on the minimum inhibitory concentration (MIC) reported in CCARM. The *S. cinnamonensis* KCTC 9708^T^ was treated at the same concentration as *S. virginiae* THA-960. 100 μL of sample was loaded on paper discs (8 mm diameter, Advantec Toyo Kaisha, Ltd., Chiyoda-ku, Tokyo, Japan). Indicators are spreading on Muller-Hinton agar (MHA, BD Difco, Franklin, NJ, USA) plate as 1×10^6^ CFU/mL. After that, the plate was incubating in 30°C for 24 h. The diameter of inhibition zone size was measured by calipers.

#### Antimicrobial bioassays and scanning electron microscope analysis

A time-kill assay was performed with MDR-*S. aureus*. In a 15 mL conical tube, 2 mL of 2 × MIC and 2 × MBC samples were mixed with 2 × 10^7^ CFU/mL MDR-*S. aureus* in nutrient broth. Controls included untreated MDR-*S. aureus*. Incubation at 37°C occurred for 0 − 24 h. Optical density was measured every 3 h using a UV spectrophotometer. After 18 h, 100 μL of a 10x diluted culture was spread on an MHA plate for colony counting. The bactericidal activity was defined as a decrease of more than 3 log_10_ in CFU/mL. To obtain the MIC and minimum bactericidal concentration (MBC) for antimicrobial activity, broth microdilution method^29^^,^^30^ was conducted by Clinical and Laboratory Standards Institute (CLSI) guidelines^4^ with four strains of *S. aureus*.

Scanning electron microscope (SEM) analysis was performed to confirm the morphological change of pathogen according to sample effects. The pathogen was *S. aureus* CCARM 3089 as known as MDR-*S. aureus*. The group treated with a sample of MIC concentration on the pathogen and the untreated group were reacted in 37°C, for 12 h. Thereafter, centrifugation is performed at 4,000 rpm, all supernatants are discarded and cells are collected. For the protein fixation, 2.5% glutaraldehyde (Daejung chemiclas & metals Co.,Ltd. Siheung-si, Gyeonggi-do, Republic of Korea) was added to cells and reacted 4°C in 2 h. After washing 3 times with PBS (Bioneer, Daejeon, Republic of Korea), 1% osmium tetroxide (Sigma Aldrich, St. Louis, MO, USA) was added to cells for lipid fixation and reacted in 4°C, for 1 h. After washing three times using PBS, the cells were reacted for 10 min in the order of 30, 50, 70, 80, 90, and 3 times of 100% ethanol (Daejung chemiclas & metals Co.,Ltd. Siheung-si, Gyeonggi-do, Republic of Korea) to conduct with the dehydration of the cells. After dehydration was finished, hexamethyldisilazane (Sigma Aldrich, St. Louis, USA) was treated for 10 min and discarded, and 100 μL was additionally treated for drying on 24 h. After 24 h, the completely dried bacteria were placed on the stub to which the carbon tape was attached, and then platinum coating was performed using an Ion sputter coater. Finally, the morphological change of bacteria was analyzed using scanning electron microscope (SU8010, Hitachi, Ltd., Chiyoda-ku, Tokyo, Japan).

#### Sequencing, genome assembly, and genome annotation

The PacBio HiFi reads were assembled by using the Microbial Genome Assembly application (SMRT Link v11.1.0.166339). The chromosome sequence was further polished by using the Illumina short reads with Pilon v1.21.^31^ Quality of the genome assembly was assessed by using Benchmarking Universal Single-Copy Orthologs (BUSCO v5.4.7) with *actinobacteria_class_odb10* lineage data.^32^ Protein-coding and RNA genes were predicted by using Prokka (v1.14.6)^33^ and RNAmmer (v1.2),^34^ respectively. Genomic features, including predicted genes and biosynthetic gene clusters (BGCs), were graphically presented by using Circos (v0.69-9).^35^ Functional annotation of Clusters of Orthologous Groups (COGs) for the predicted genes was performed using eggNOG-mapper.^36^ The prediction of BGCs in genome sequences was performed using antiSMASH (v6.1.1).^37^

#### Phylogenetic and phylogenomic analyses for species identification

Species identification utilized the consensus sequence (1,512 bp) derived from seven predicted 16S rRNA gene sequences in the genome of strain THA-960. The consensus sequence was obtained by using EMBOSS Cons web server.^38^ The sequence was analyzed using the EZBioCloud database (version 2023.06.29),^39^ and hits of the sequence were extracted for further analysis (Table S2). The 16S rDNA sequences of THA-960 and 50 hits were aligned using the MUSCLE algorithm with default parameters. Subsequently, overhang regions were trimmed, resulting in a final alignment spanning 1,377 base pairs including gaps. A phylogenetic tree based on the 16S rDNA sequence alignment was constructed using the maximum likelihood method under the Tamura-Nei model, incorporating a discrete gamma distribution and invariable sites model (TN93+G+I), implemented in Molecular Evolutionary Genetics Analysis – Computational Core (MEGA-CC v10.2.6).^40^ For the phylogenomic analysis, 2,817 *Streptomyces* genomes were obtained from NCBI RefSeq database (accessed on June 18, 2023). Average nucleotide identity values between strain THA-960 and each of the 2,817 genomes were calculated by Orthologous Average Nucleotide Identity Tool (OrthoANI v1.40).^41^ In addition, the value of DNA-DNA relatedness was obtained by calculation of digital DNA-DNA hybridization (dDDH) under the recommended setting of Genome-to-Genome Distance Calculator (GGDC v3.0).^42^ The dDDH values were calculated with 520 selected genomes (Table S3). Visualization of the dDDH and OrthoANI calculations was achieved by generating a scatter plot using the R programming language (v4.2.3) with the *ggplot2*, *ggExtra*, and *ggthemes* packages.^43^^,^^44^^,^^45^^,^^46^

#### Metabolomic analysis

The analysis was performed using an UPLC (Ultimate 3000, Thermo Scientific, Waltham, MA, USA) connected with Triple TOF 5600+ mass spectrometer detector (AB Sciex, Framingham, MA, USA). The column was used Waters Cortex C8 (2.1 × 150 mm, 1.6 μm). Cell pellets were extracted with acetone to detect amycomicin, while the culture supernatant was analyzed directly. The mass spectrometer was operated in positive and negative electrospray ionization (ESI) mode with full scan and information dependent acquisition scan methods, with a range of 100 – 2,000 Da. 0.1% formic acid in water and 0.1% formic acid in acetonitrile were used as the mobile phases A and B. The chromatographic separation of gradient elution at a flow rate was 0.25 mL/min. The gradient elution condition was followed: 0 – 0.1 min, 0% B; 0.1 – 20 min, 0 – 20% B; 20 – 48 min, 20 – 100% B; 48 – 53 min, 100% B; 53 – 53.1 min, 100 – 0% B; 53.1 – 60 min, 0% B. The energies of the MS low and high collisions were 10 and 35 to 50 eV. The drying gas was used nitrogen. The nebulizing gas pressure was 50 psi, heating gas pressure was 50 psi, and curtain gas pressure was 25 psi. The desolvation temperature was 500°C and column temperature was 45°C. The capillary and sampling cones were used 5.5 KV (positive) and 4.5 KV (negative).

#### Structural prediction of protein-ligand complexes

To investigate the binding modes of amycomicin with various target proteins, three-dimensional complex structures were predicted using AlphaFold3 (AF3).^47^ The protein sequences were retrieved from the UniProt database using their respective accession numbers. For each prediction, the chemical structure of amycomicin was provided to a standalone server alongside the protein sequence. The model with the highest interface predicted Template Modeling score (ipTM) and predicted Local Distance Difference Test (pLDDT) was selected for downstream analysis to ensure the reliability of the predicted binding interface. The confidence of the predicted complexes was evaluated using multiple internal metrics from AF3. Global structural accuracy was assessed via the pTM score, while the precision of the protein-ligand interaction was evaluated by using the ipTM score. Local atom-level confidence was determined by the pLDDT values, with a focus on the interface pLDDT to validate the stability of the binding pocket. Additionally, Predicted Aligned Error (PAE) values, both overall and interface-specific, were analyzed to assess the relative positional certainty of the ligand within the binding site.

Characterization of the chemical interactions between amycomicin and the predicted protein targets was performed using the Protein-Ligand Interaction Profiler (PLIP).^55^ The tool was utilized to detect and quantify various non-covalent interactions, including hydrogen bonds, hydrophobic interactions, and salt bridges. General spatial contacts were also retrieved to provide a comprehensive map of the interaction network within the binding pocket. The theoretical binding affinity of the predicted complexes was estimated using PRODIGY-LIG.^56^ This method calculates the Gibbs free energy of binding (*ΔG*) based on the contact residues and the structural properties of the protein-ligand interface. The predicted *ΔG* values (kcal/mol) were used to rank the potential targets and evaluate the thermodynamic favorability of the amycomicin-protein interactions.

### Quantification and statistical analysis

#### Bioinformatics analysis

To visualize the relationship between strain THA-960 and the 520 *Streptomyces* spp., a phylogenomic tree was constructed using the standalone version of Composition Vector Tree (CVTree).^48^ The proteome sequence of *Escherichia coli* str. K-12 substr. MG1655 (GCF_000005845.2) was used as an out-group for the phylogenomic tree. A *K*-tuple length of 6 was chosen for CVTree, as it has been suggested as the optimal value for bacterial phylogeny.^49^ Sequence homology of biosynthetic gene clusters registered in Minimum Information about a Biosynthetic Gene cluster database (v3.1)^50^ was searched by using protein sequences against genome sequences of THA-960 and the 520 *Streptomyces* spp. The phylogenomic tree was visually presented with the homology distribution of protein sequences by using Graphical Phylogenetic Analysis (GraPhlAn v1.1.4).^51^ Nucleotide sequences of predicted BGCs were analyzed by biosynthetic gene similarity clustering and prospecting engine (BiG-SCAPE).^52^ The resulting networks of predicted BGCs for non-ribosomal peptide synthetase (NRPS) and polyketide synthase (PKS) type I were visualized by using Cytoscape (v3.10.0).^53^

To assess the bioactivity potential of BGCs, predictions were performed using NPBDetect (v1.0), a neural network-based tool that estimates the likelihood of eight bioactivity classes, including antibacterial, antifungal, antitumor/cytotoxic, and siderophore activities, from antiSMASH-generated GenBank files. BGC sequences were obtained from antiSMASH (v8.0.4), and NPBDetect was executed in high-confidence mode. Prediction outputs were returned as probability scores for each bioactivity class, with values ≥ 0.5 considered positive predictions.^54^

#### Statistical analysis

Data are presented as mean ± standard deviation (SD) from three independent experiments. Group differences were evaluated using two-way analysis of variance (ANOVA) followed by Dunnett’s multiple comparisons test in GraphPad Prism version 10. Statistical significance was defined as ∗*P* < 0.05, ∗∗*P* < 0.01, ∗∗∗*P* < 0.001, and ∗∗∗∗*P* < 0.0001, and assumptions of normal distribution were verified before performing the analyses.

## Supplemental information

Document S1. Figures S1–S8 and Tables S1–S6

Data S1. List of 520 Streptomyces genomes used in the phylogenomic analysis
