# Supplementary material for: Genome-guided discovery of antibiotic activity in Streptomyces virginiae THA-960 against multidrug resistant Staphylococcus aureus
Source: iScience. 2026 Jun 9;29(6):116311. doi: 10.1016/j.isci.2026.116311 (PMC13273461; doi:10.1016/j.isci.2026.116311)
Supplement: Document S1. Figures S1–S8 and Tables S1–S6 [file mmc1.pdf]

iScience, Volume *29*

Supplemental information

Genome-guided discovery of antibiotic activity in *Streptomyces virginiae* THA-960 against multidrug resistant *Staphylococcus aureus*

Trang Thi Minh Nguyen, Jeyong Jung, Xiangji Jin, Qiwen Zheng, Jaeyoung Choi, and Tae-Hoo Yi

Supplementary Figures + legends

Figure S1. Phylogenetic tree constructed based on the analysis of 16S rRNA gene sequences. The tree represents the evolutionary relationships of strain THA-960 and 49 closely related species shown in Table S1. Bootstrap values below 50% were not shown in the figure. Bootstrap values below 50% were not shown. Phylogenetic analysis was performed using MEGA-CC under TN93+G+I model as described in STAR Methods.

Figure S2. Distribution of proteins in gene cluster for virginiamycin S1 (BGC0001116) across genome sequences of strain THA-960 and 520 selected *Streptomyces* strains. Protein sequences of each cluster were searched using TBLATN with an E-value cutoff of 1e-5. The intensity of the dark navy-blue color in each cell represents the maximum bit score for the corresponding protein, indicating the level of homology. A darker color indicates a higher degree of sequence homology. The strain THA-960 is denoted by a filled-star mark at the terminal node. Protein homology was determined using TBLASTN with an E-value cutoff of 1e-5, and intensity of dark navy-blue cells represents maximum bit scores, as detailed in STAR Methods.

Figure S3. Genomic relatedness of strain THA-960 with the six *S. virginiae* genomes. Pairwise OrthoANI calculations were performed by using genome sequences of strain THA-960 and the six *S. virginiae* strains that did not show significant genomic identity to strain THA-960. Accessions for the six *S. virginiae* genomes: GCF_026342285.1 (strain NBC_00276), GCF_001270565.1 (NRRL B-1447), GCF_026341715.1 (NBC_00640), GCF_026342475.1 (NBC_00227), GCF_026340475.1 (NBC_01311), and GCF_000716685.1 (NRRL B-8091). Pairwise OrthoANI and dDDH calculations were performed, and genome accessions are provided.

Figure S4. Distribution of proteins in gene clusters (Clusters 1–4) well conserved in *Streptomyces virginiae* across genome sequences of strain THA-960 and 521 selected

***Streptomyces* strains.** Protein sequences of each cluster were searched using TBLATN with an E-value cutoff of 1e-5. The intensity of the dark navy-blue color in each cell represents the maximum bit score for the corresponding protein, indicating the level of homology. A darker color indicates a higher degree of sequence homology. The strain THA-960 is denoted by a filled-star mark at the terminal node.

Figure S5. Distribution of proteins in gene clusters (Clusters 5–8) well conserved in *Streptomyces virginiae* across genome sequences of strain THA-960 and 521 selected *Streptomyces* strains. Protein sequences of each cluster were searched using TBLATN with an E-value cutoff of 1e-5. The intensity of the dark navy-blue color in each cell represents the maximum bit score for the corresponding protein, indicating the level of homology. A darker color indicates a higher degree of sequence homology. The strain THA-960 is denoted by a filled-star mark at the terminal node. Homology searches were performed using TBLASTN, and the degree of conservation is reflected by cell color intensity, as described in STAR Methods.

Figure S6. A magnified view of the phylogenomic tree encompassing the strains belonging to the Groups 1-3. Genome accessions for the Group 1-3 strains were shown with a phylogenomic subtree. *S. nojiriensis* JCM 3382 was also indicated in the tree with the bold face. Genome accessions are indicated, and tree construction followed the CVTree method as detailed in STAR Methods.

Figure S7. Sequence similarity network of NRPS clusters generated using BiG-SCAPE. The network encompasses 3,838 BGCs derived from the analysis of 521 selected *Streptomyces* genomes and MIBiG database. Nodes in the network were color-coded based on their taxonomic groups or origin, which were shown at the bottom of the figure. The edges connecting the nodes are represented by a color gradient, reflecting the raw distances obtained

from the BiG-SCAPE analysis. Components containing BGCs predicted to produce streptothricin and tambromycin were indicated by dashed boxes.

Figure S8. Distribution of biosynthetic gene clusters well conserved in *S. virginiae* genomes. Protein sequences of each cluster were searched using TBLATN with an E-value cutoff of 1e-5 against genome sequences of strain THA-960 and 520 selected *Streptomyces* strains. The intensity of the dark navy-blue color in each cell represents the maximum bit score for the corresponding protein, indicating the level of homology. A darker color indicates a higher degree of sequence homology. The strain THA-960 is denoted by a filled-star mark at the terminal node. Representative clusters include BGC0000100 (Monensin), BGC0000233 (Hedamycin (LC-MS/MS)), BGC0000303 (Anthramycin (LC-MS/MS)), BGC0000432 (Streptothricin), BGC0000841 (Alanylclavam_01 (LC-MS/MS)), BGC0000843 (Alanylclavam_02 (LC-MS/MS)).

Supplementary Figures

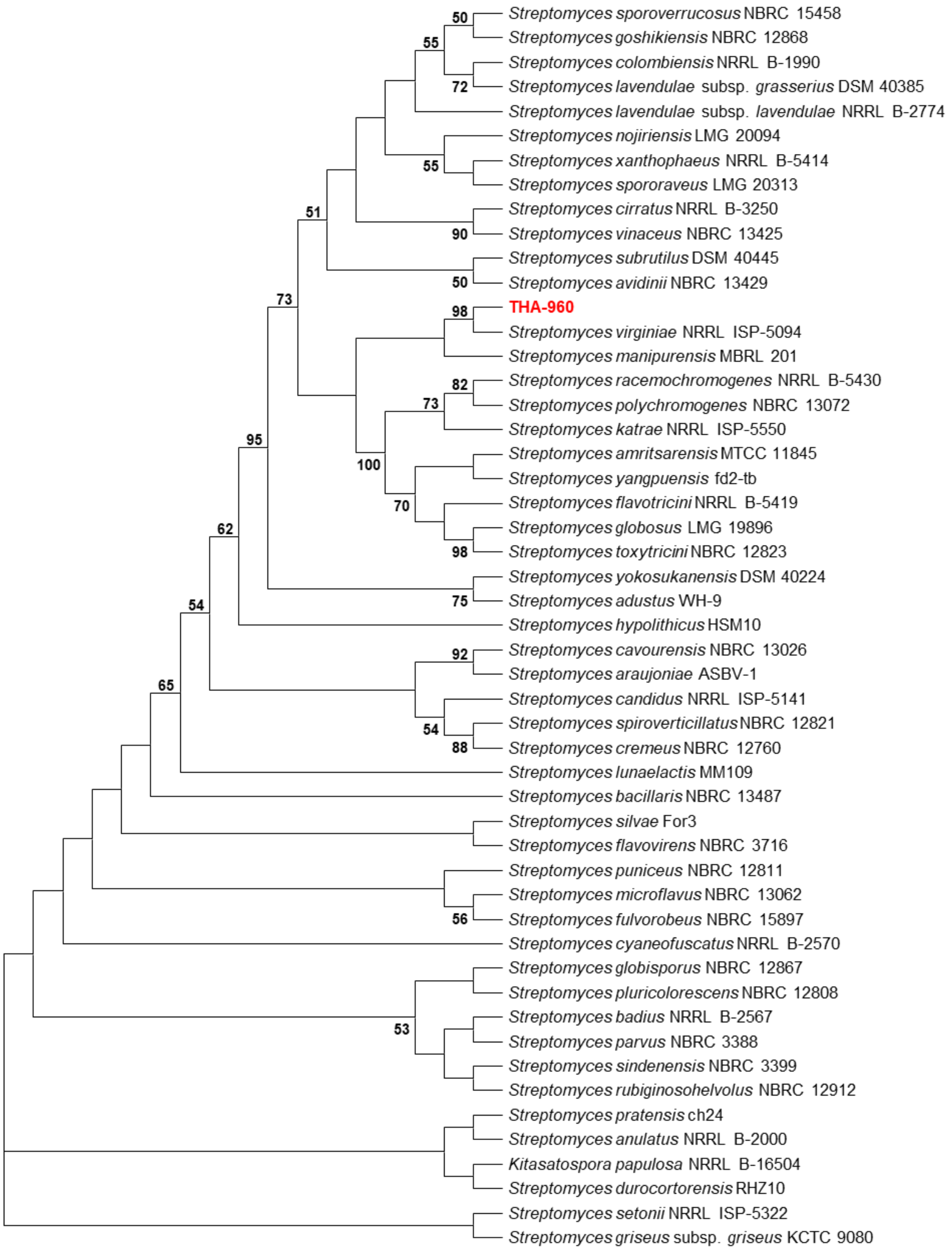

Figure S1. Phylogenetic tree constructed based on the analysis of 16S rRNA gene sequences. The tree represents the evolutionary relationships of strain THA-960 and 49 closely related species shown in Table S1. Bootstrap values below 50% were not shown in the figure.

Bootstrap values below 50% were not shown in the figure. Bootstrap values below 50% were not shown. Phylogenetic analysis was performed using MEGA-CC under TN93+G+I model as described in STAR Methods.

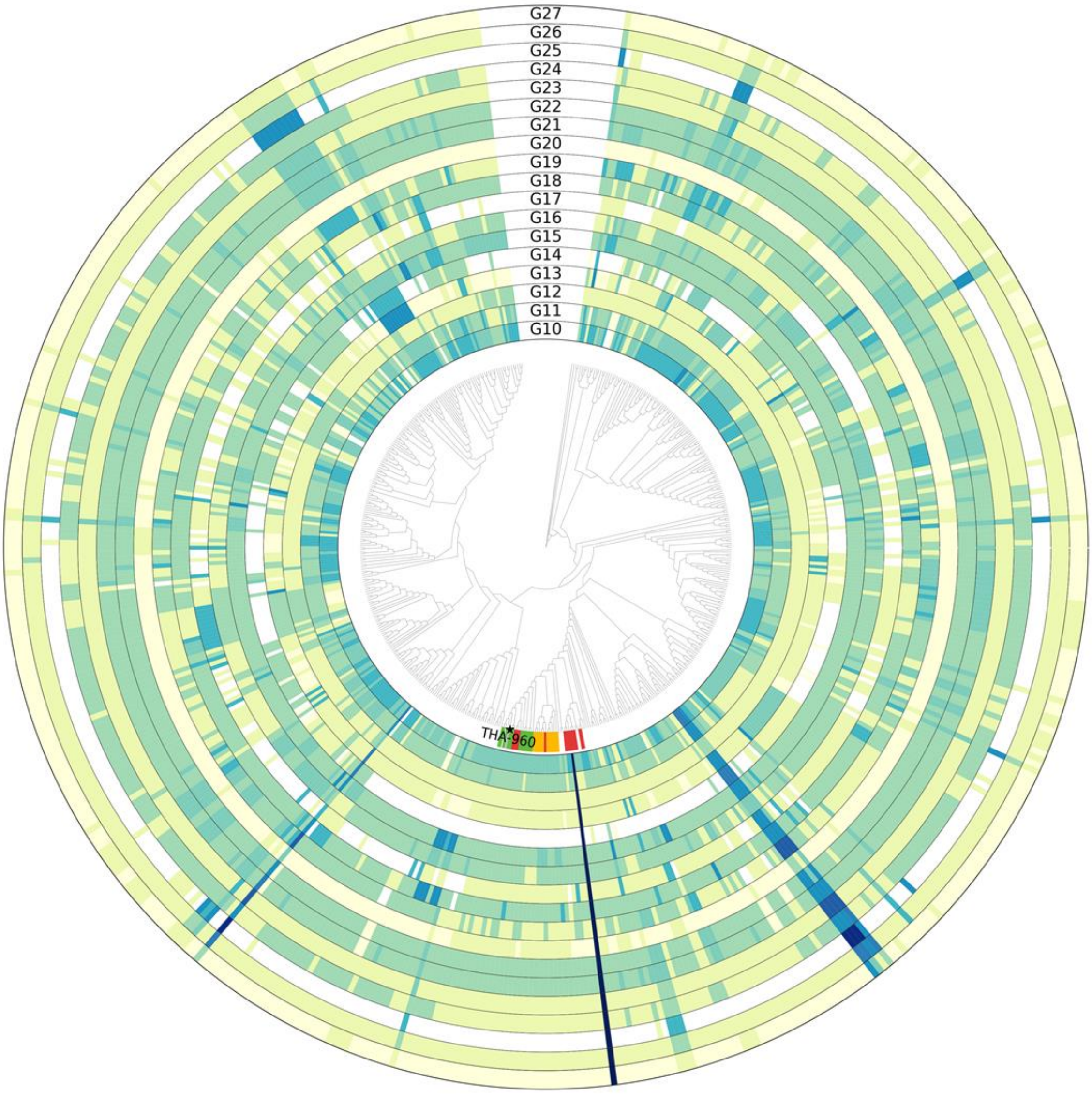

Figure S2. Distribution of proteins in gene cluster for virginiamycin S1 (BGC0001116) across genome sequences of strain THA-960 and 520 selected *Streptomyces* strains. Protein sequences of each cluster were searched using TBLATN with an E-value cutoff of 1e-5. The intensity of the dark navy-blue color in each cell represents the maximum bit score for the corresponding protein, indicating the level of homology. A darker color indicates a higher

degree of sequence homology. The strain THA-960 is denoted by a filled-star mark at the terminal node. Protein homology was determined using TBLASTN with an E-value cutoff of 1e-5, and intensity of dark navy-blue cells represents maximum bit scores, as detailed in STAR Methods.

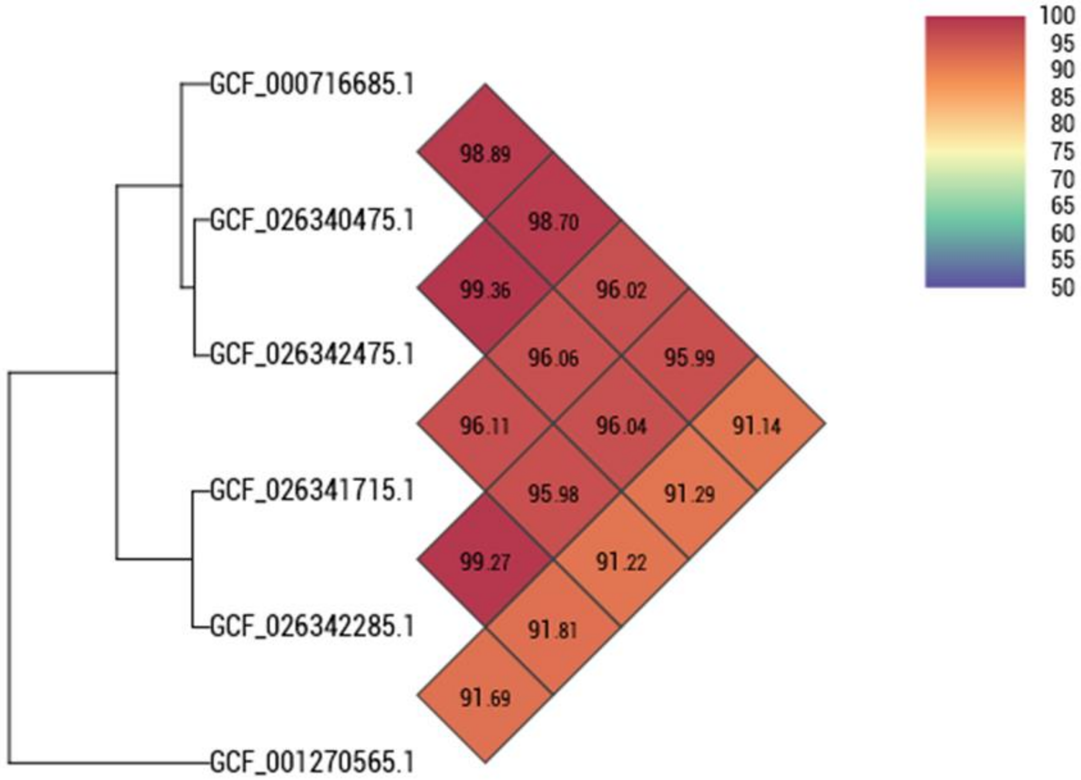

Figure S3. Genomic relatedness of strain THA-960 with the six *S. virginiae* genomes. Pairwise OrthoANI calculations were performed by using genome sequences of strain THA-960 and the six *S. virginiae* strains that did not show significant genomic identity to strain THA-960. Accessions for the six *S. virginiae* genomes: GCF_026342285.1 (strain NBC_00276), GCF_001270565.1 (NRRL B-1447), GCF_026341715.1 (NBC_00640), GCF_026342475.1 (NBC_00227), GCF_026340475.1 (NBC_01311), and GCF_000716685.1 (NRRL B-8091). Pairwise OrthoANI and dDDH calculations were performed, and genome accessions are provided.

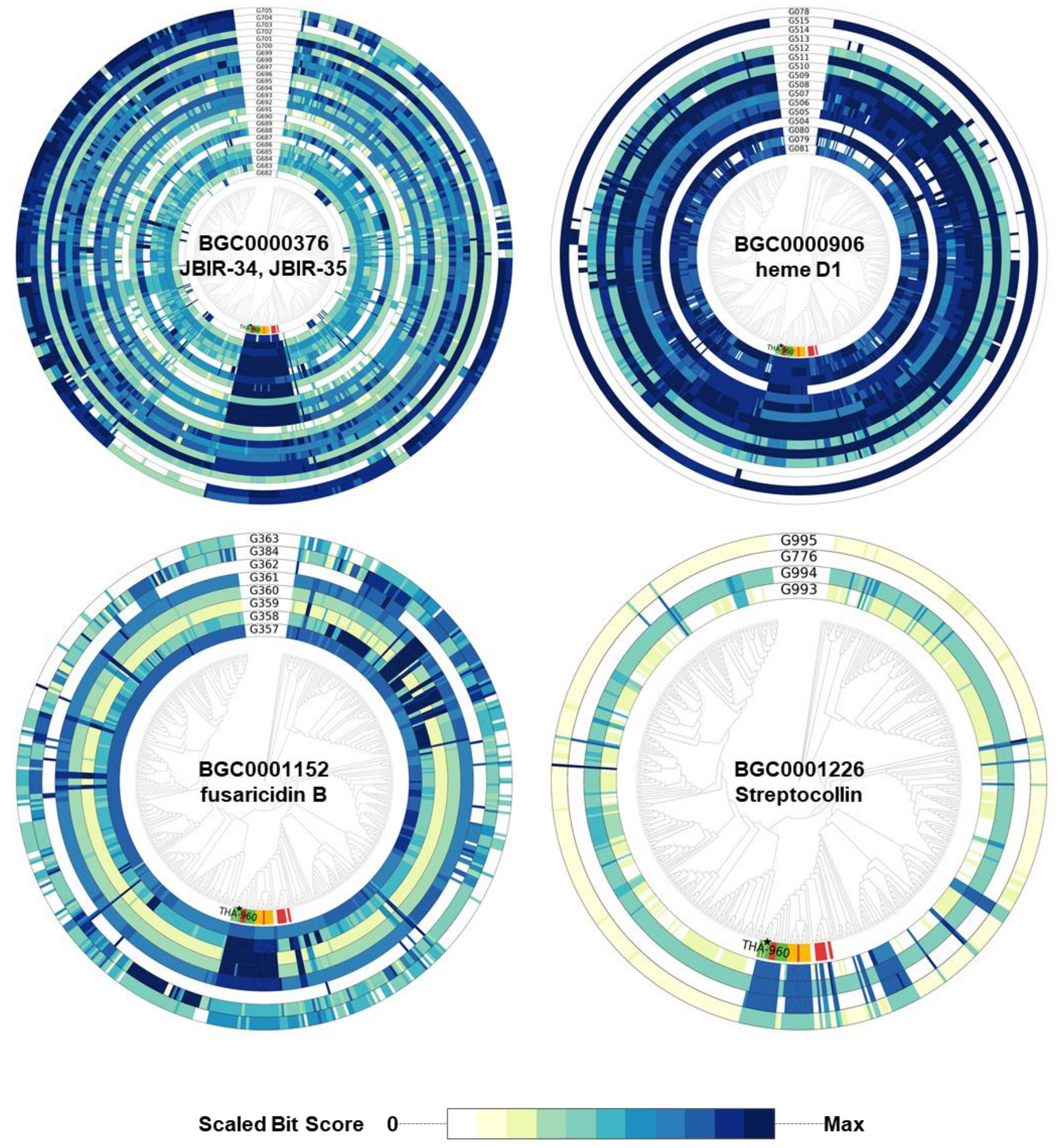

Figure S4. Distribution of proteins in gene clusters (Clusters 1–4) well conserved in *Streptomyces virginiae* across genome sequences of strain THA-960 and 521 selected *Streptomyces* strains. Protein sequences of each cluster were searched using TBLATN with an E-value cutoff of 1e-5. The intensity of the dark navy-blue color in each cell represents the maximum bit score for the corresponding protein, indicating the level of homology. A darker color indicates a higher degree of sequence homology. The strain THA-960 is denoted by a filled-star mark at the terminal node.

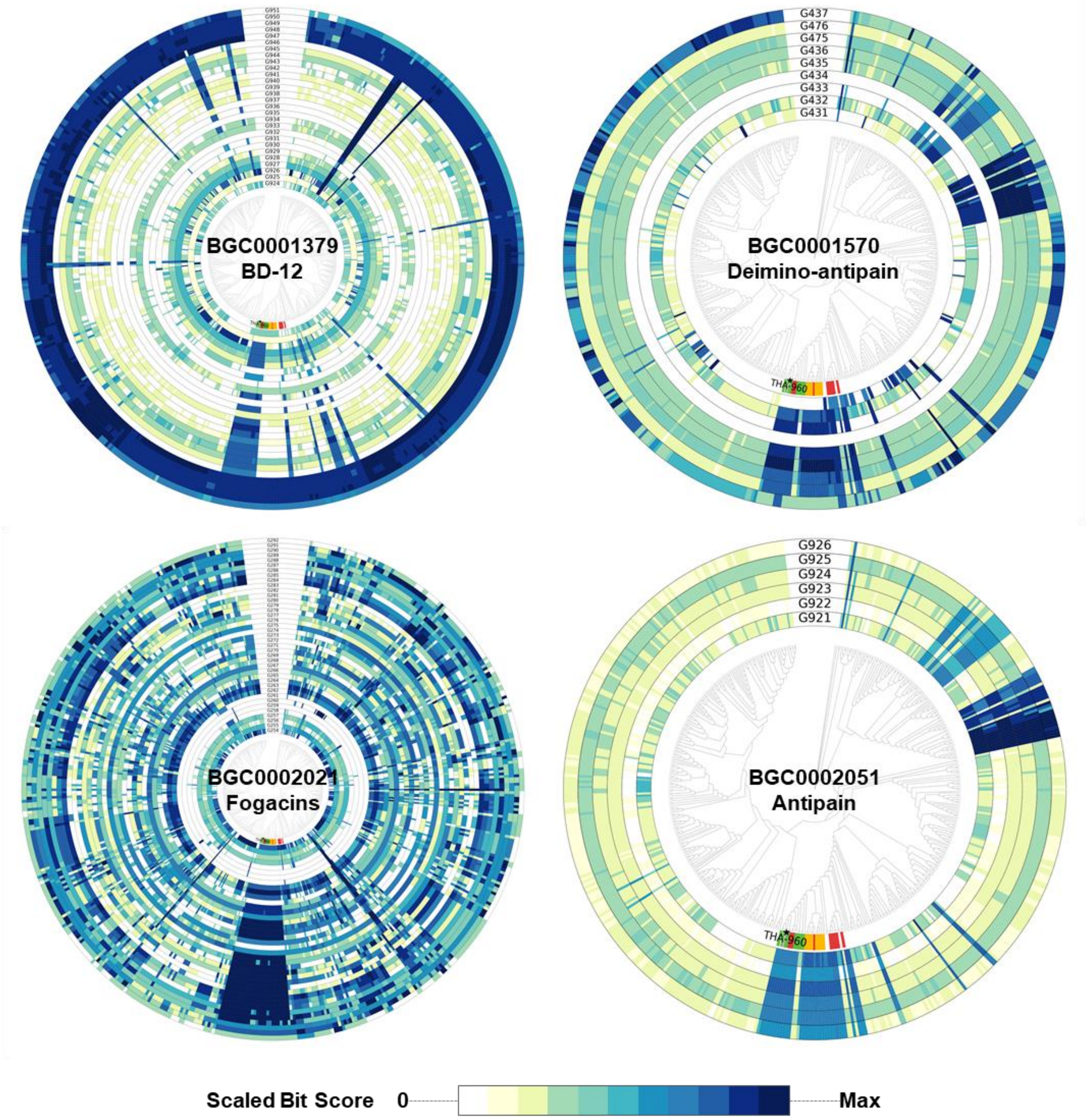

Figure S5. Distribution of proteins in gene clusters (Clusters 5–8) well conserved in *Streptomyces virginiae* across genome sequences of strain THA-960 and 521 selected *Streptomyces* strains. Protein sequences of each cluster were searched using TBLATN with an E-value cutoff of 1e-5. The intensity of the dark navy-blue color in each cell represents the maximum bit score for the corresponding protein, indicating the level of homology. A darker color indicates a higher degree of sequence homology. The strain THA-960 is denoted by a filled-star mark at the terminal node.

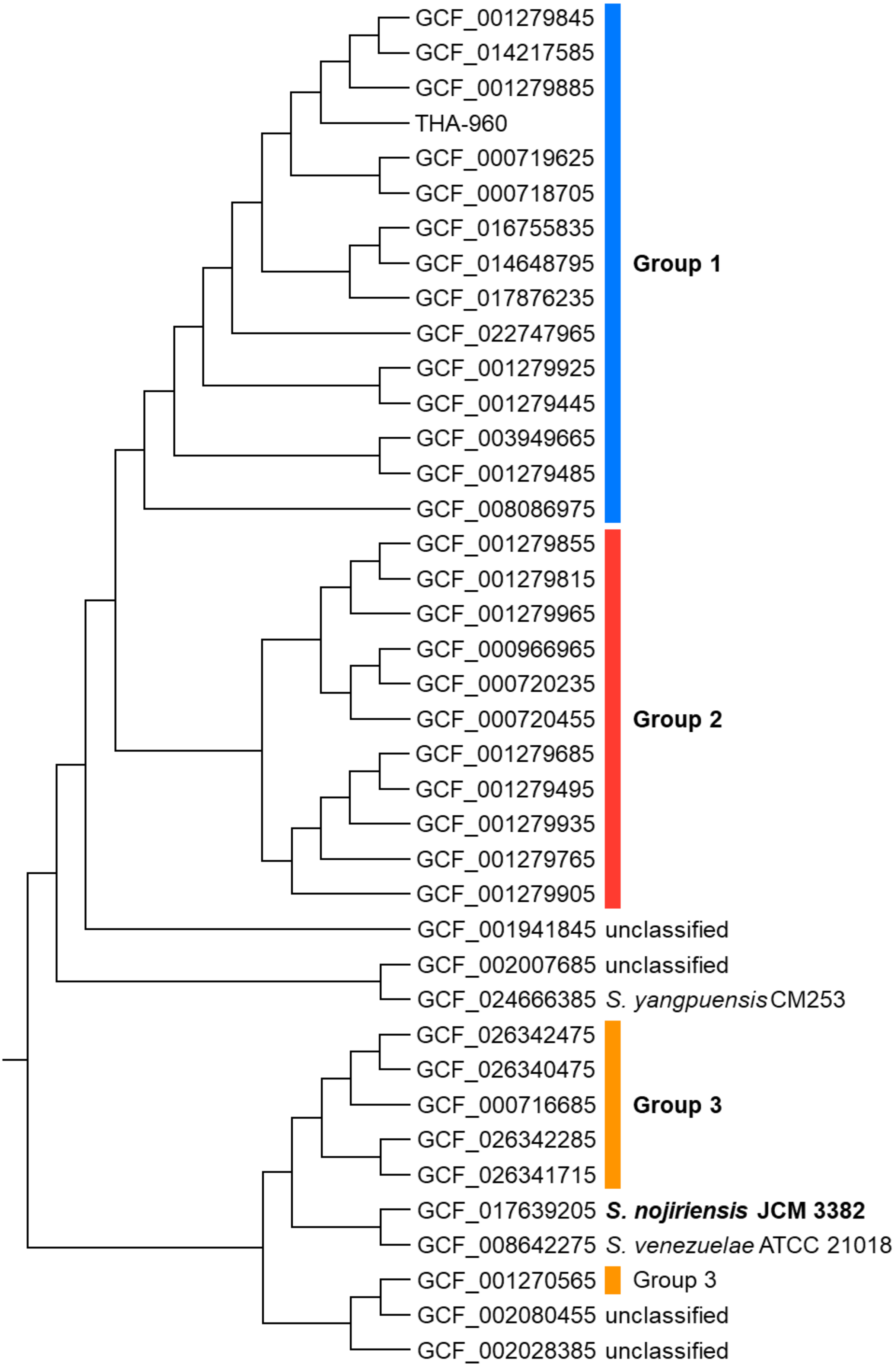
GCF_001279845
GCF_014217585
GCF_001279885
THA-960
GCF_000719625
GCF_000718705
GCF_016755835
GCF_014648795
GCF_017876235
GCF_022747965
GCF_001279925
GCF_001279445
GCF_003949665
GCF_001279485
GCF_008086975
Group 1
GCF_001279855
GCF_001279815
GCF_001279965
GCF_000966965
GCF_000720235
GCF_000720455
GCF_001279685
GCF_001279495
GCF_001279935
GCF_001279765
GCF_001279905
Group 2
GCF_001941845 unclassified
GCF_002007685 unclassified
GCF_024666385 S. yangpuensis CM253
GCF_026342475
GCF_026340475
GCF_000716685
GCF_026342285
GCF_026341715
Group 3
GCF_017639205 S. nojiriensis JCM 3382
GCF_008642275 S. venezuelae ATCC 21018
GCF_001270565 Group 3
GCF_002080455 unclassified
GCF_002028385 unclassified

Figure S6. A magnified view of the phylogenomic tree encompassing the strains belonging to the Groups 1-3. Genome accessions for the Group 1-3 strains were shown with a phylogenomic subtree. *S. nojiriensis* JCM 3382 was also indicated in the tree with the bold face. Genome accessions are indicated, and tree construction followed the CVTree method as detailed in STAR Methods.

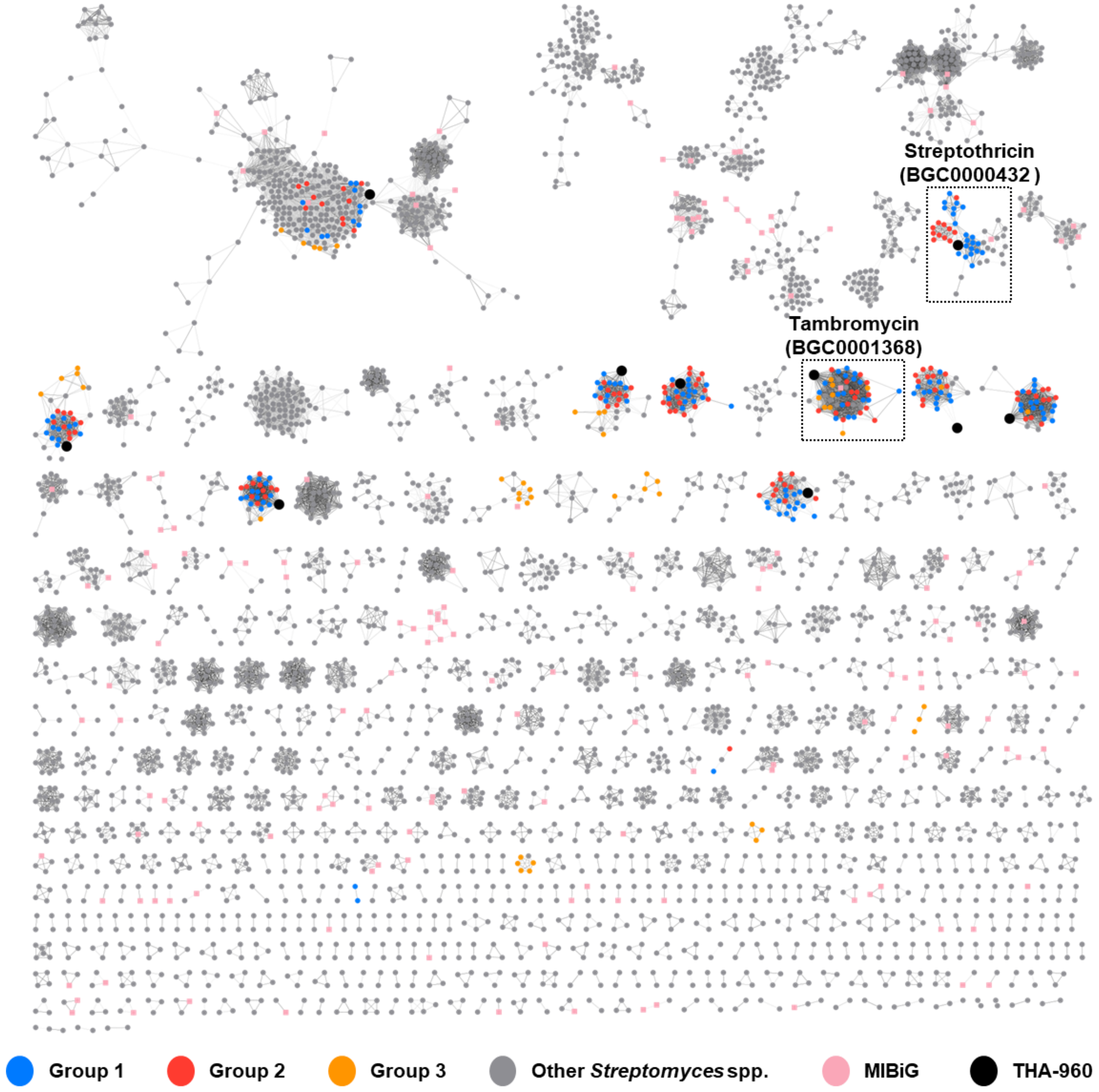

Figure S7. Sequence similarity network of NRPS clusters generated using BiG-SCAPE. The network encompasses 3,838 BGCs derived from the analysis of 521 selected *Streptomyces* genomes and MIBiG database. Nodes in the network were color-coded based on their taxonomic groups or origin, which were shown at the bottom of the figure. The edges connecting the nodes are represented by a color gradient, reflecting the raw distances obtained from the BiG-SCAPE analysis. Components containing BGCs predicted to produce streptothricin and tambromycin were indicated by dashed boxes.

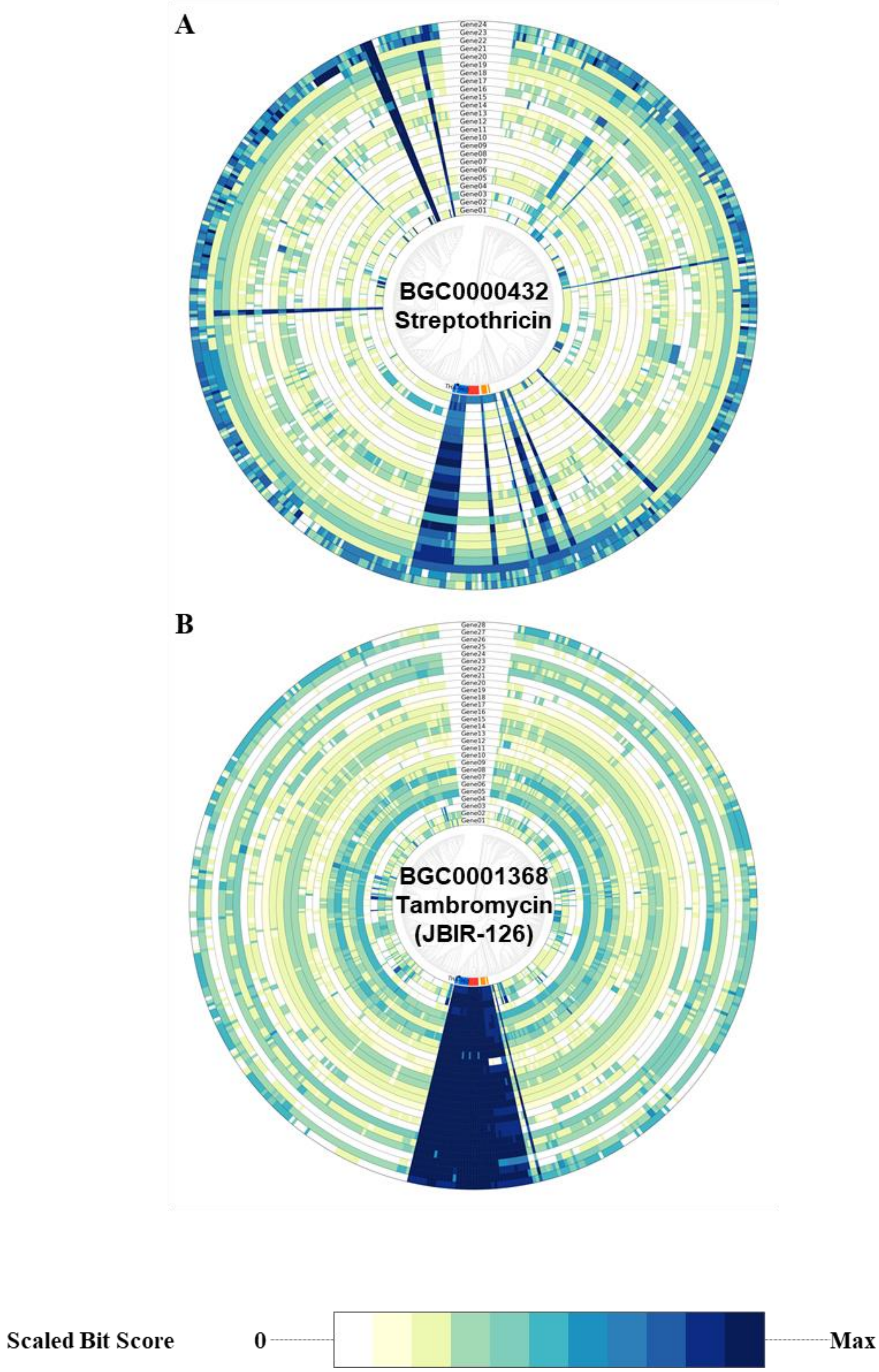

Figure S8. Distribution of biosynthetic gene clusters well conserved in *S. virginiae* genomes. Protein sequences of each cluster were searched using TBLATN with an E-value cutoff of 1e-5 against genome sequences of strain THA-960 and 520 selected *Streptomyces* strains. The intensity of the dark navy-blue color in each cell represents the maximum bit score

for the corresponding protein, indicating the level of homology. A darker color indicates a higher degree of sequence homology. The strain THA-960 is denoted by a filled-star mark at the terminal node. Representative clusters include BGC0000100 (Monensin), BGC0000233 (Hedamycin (LC-MS/MS)), BGC0000303 (Anthramycin (LC-MS/MS)), BGC0000432 (Streptothricin), BGC0000841 (Alanylclavam_01 (LC-MS/MS)), BGC0000843 (Alanylclavam_02 (LC-MS/MS)).

Supplementary Tables

Table S1. List of multidrug-resistant bacteria and antimicrobial activity of THA-960 culture filtrate

Table S2. Hits of the 16S rRNA gene sequence of strain THA-960 against EzBioCloud database. The 16S rRNA gene sequences were aligned using MUSCLE, and phylogenetic relationships were inferred as detailed in STAR Methods.

Table S3. Functional annotation of predicted proteins in strain THA-960 cased on Clusters of Orthologous Groups (COGs) classification. Annotation was performed using eggNOG-mapper (v2.1.13) as described in STAR Methods.

Table S4. List of predicted biosynthetic gene clusters from the genome sequence of strain THA-960. Biosynthetic gene clusters were predicted using antiSMASH (v8.0.4).

Table S5. List of *S. virginiae* genomes available from NCBI genome database and average nucleotide identity (ANI) match details. Comparisons were performed using OrthoANI (v1.40) as described in STAR Methods.

Table S6. Computational evaluation of amycomicin binding interfaces including structural confidence, interaction profiling, and predicted binding affinity using AlphaFold3.

Table S1. List of multidrug-resistant bacteria and antimicrobial activity of THA-960 culture filtrate

Indicators	Medium*	Antibiotic resistance**	Inhibition zone (mm)
Enterococcus faecalis CCARM 5171	MRS	Amp, Nor, GM, Van	13.5
Enterococcus faecium CCARM 5262	MRS	Amp, Nor, GM, Van	14.5
Staphylococcus aureus CCARM 0204	NB	Amp, Nor, GM	19
Staphylococcus aureus CCARM 0205	NB	Amp, Nor, GM	19.5
Staphylococcus aureus CCARM 3855	NB	Amp, Nor, GM	17
Staphylococcus aureus CCARM 3089	NB	Nor, Oxa, Van	15
Escherichia coli DC 0 CCARM 0237	TSB	Amp, Nor, GM	14.5
Escherichia coli DC 2 CCARM 0238	TSB	Amp, Nor, GM	14.5
Escherichia coli TEM CCARM 0235	TSB	Amp, Nor, GM	13
Escherichia coli 1507 CCARM 0236	TSB	Amp, Nor, GM	13
Pseudomonas aeruginosa CCARM 0223	NB	Amp, Nor, GM	9.5
Pseudomonas aeruginosa CCARM 0224	NB	Amp, Nor, GM	11.5
Salmonella enterica subsp. *enterica* serovar *typhimurium* CCARM 0240	NB	Amp, Nor, GM	15.5
Klebsiella oxytoca CCARM 0248	NB	Amp, Nor, GM	15
Klebsiella aerogenes 1522E CCARM 0249	NB	Amp, Nor, GM	15
Enterobacter cloacae P 99 CCARM 0252	NB	Amp, Nor, GM	15
Enterobacter cloacae 1321E CCARM 0253	NB	Amp, Nor, GM	17

*MRS, Lactobacilli MRS broth; TSB, Tryptone Soy Broth; NB Nutrient broth.
**Amp, Ampicillin; Nor, Norfloxacin; GM, Gentamycin; Oxa, Oxacillin; Van, Vancomycin.

Table S2. Hits of the 16S rRNA gene sequence of strain THA-960 against EzBioCloud database. The 16S rRNA gene sequences were aligned using MUSCLE, and phylogenetic relationships were inferred as detailed in STAR Methods.

A*	B*	C*	D*	E*	F*
1	*Streptomyces virginiae*	NRRL ISP-5094	JOAK01000082	100	100
2	*Streptomyces xanthophaeus*	NRRL B-5414	JOFT01000080	99.72337	100
3	*Streptomyces nojiriensis*	LMG 20094	AJ781355	99.72337	100
4	*Streptomyces spororaveus*	LMG 20313	AJ781370	99.72337	100
5	*Streptomyces lavendulae* subsp. *lavendulae*	NRRL B-2774	JOEW01000098	99.65422	100
6	*Streptomyces manipurensis*	MBRL 201	JN560156	99.65398	100
7	*Streptomyces cirratus*	NRRL B-3250	AY999794	99.58506	100
8	*Streptomyces vinaceus*	NBRC 13425	AB184394	99.58333	99.58535
9	*Streptomyces sporoverrucosus*	NBRC 15458	AB184684	99.58275	99.44713
10	*Streptomyces goshikiensis*	NBRC 12868	AB184204	99.5813	99.10159
11	*Streptomyces colombiensis*	NRRL B-1990	DQ026646	99.51591	100
12	*Streptomyces subrutilus*	DSM 40445	X80825	99.44637	100
13	*Streptomyces avidinii*	NBRC 13429	AB184395	99.44637	99.93089

14	*Streptomyces flavotricini*	NRRL B-5419	JNXV01000042	99.17012	100
15	*Streptomyces amritsarensis*	MTCC 11845	MQUR01000179	99.17012	100
16	*Streptomyces yangpuensis*	fd2-tb	LBMK01000002	99.17012	100
17	*Streptomyces racemochromogenes*	NRRL B-5430	DQ026656	99.10097	100
18	*Streptomyces polychromogenes*	NBRC 13072	AB184292	99.09722	99.58564
19	*Streptomyces globosus*	LMG 19896	AJ781330	98.75519	100
20	*Streptomyces toxytricini*	NBRC 12823	AB184173	98.7526	99.79282
21	*Streptomyces cavourensis*	NBRC 13026	AB184264	98.68512	99.93103
22	*Streptomyces spiroverticillatus*	NBRC 12821	AB249921	98.61687	100
23	*Streptomyces pratensis*	ch24	JQ806215	98.61213	94.62069
24	*Streptomyces badius*	NRRL B-2567	AY999783	98.54772	100
25	*Streptomyces yokosukanensis*	DSM 40224	KQ948269	98.54772	100
26	*Streptomyces katrae*	NRRL ISP-5550	JZWV01000648	98.54772	100
27	*Streptomyces candidus*	NRRL ISP-5141	DQ026663	98.54671	100
28	*Streptomyces globisporus*	NBRC 12867	AB184203	98.54571	99.93103
29	*Streptomyces sindenensis*	NBRC 3399	AB184759	98.54571	99.93103
30	*Streptomyces parvus*	NBRC 3388	AB184756	98.54571	99.86207
31	*Streptomyces pluricolorescens*	NBRC 12808	AB184162	98.54268	99.65517
32	*Streptomyces rubiginosohelvolus*	NBRC 12912	AB184240	98.53964	99.44828
33	*Streptomyces cremeus*	NBRC 12760	AB184124	98.53862	99.58564
34	*Streptomyces setonii*	NRRL ISP-5322	MUNB01000146	98.47856	100
35	*Streptomyces anulatus*	NRRL B-2000	DQ026637	98.47856	100
36	*Kitasatospora papulosa*	NRRL B-16504	JNYQ01000038	98.47856	100
37	*Streptomyces adustus*	WH-9	LC026279	98.47856	100
38	*Streptomyces microflavus*	NBRC 13062	AB184284	98.47751	100
39	*Streptomyces puniceus*	NBRC 12811	AB184163	98.47751	99.93094
40	*Streptomyces fulvorobeus*	NBRC 15897	AB184711	98.4754	99.86188
41	*Streptomyces durocortorensis*	RHZ10	MW582863	98.42294	96.4779
42	*Streptomyces lunaelactis*	MM109	KM207217	98.378	98.06897
43	*Streptomyces silvae*	For3	MW479423	98.37571	97.92818
44	*Streptomyces flavovirens*	NBRC 3716	AB184834	98.34025	100
45	*Streptomyces cyaneofuscatus*	NRRL B-2570	JOEM01000050	98.34025	100
46	*Streptomyces griseus* subsp. *griseus*	KCTC 9080	M76388	98.33795	100
47	*Streptomyces araujoniae*	ASBV-1	EU792889	98.30986	98.20442
48	*Streptomyces lavendulae* subsp. *grasserius*	DSM 40385	AY999841	98.28694	97.37388
49	*Streptomyces hypolithicus*	HSM10	EU196762	98.2699	100
50	*Streptomyces bacillaris*	NBRC 13487	AB184439	98.2699	99.93103

*A: Rank, B: Name, C: Strain, D: Accession, E: Pairwise Similarity (%), and F: Completeness (%).

Table S3. Functional annotation of predicted proteins in strain THA-960 cased on Clusters of Orthologous Groups (COGs) classification. Annotation was performed using eggNOG-mapper (v2.1.13) as described in STAR Methods.

COG	Functional category	Count	Ratio (%)
A	RNA processing and modification	1	0.0137
B	Chromatin structure and dynamics	2	0.0275
C	Energy production and conversion	316	4.3407
D	Cell cycle control, cell division, chromosome partitioning	31	0.4258
E	Amino acid transport and metabolism	390	5.3571
F	Nucleotide transport and metabolism	94	1.2912
G	Carbohydrate transport and metabolism	316	4.3407
H	Coenzyme transport and metabolism	150	2.0604
I	Lipid transport and metabolism	208	2.8571
J	Translation, ribosomal structure and biogenesis	185	2.5412
K	Transcription	543	7.4588
L	Replication, recombination and repair	187	2.5687
M	Cell wall/membrane/envelope biogenesis	212	2.9121
N	Cell motility	0	0.0000
O	Posttranslational modification, protein turnover, chaperones	169	2.3214
P	Inorganic ion transport and metabolism	266	3.6538
Q	Secondary metabolites biosynthesis, transport and catabolism	196	2.6923
R	General function prediction only	1,278	17.5549
S	Function unknown	2,219	30.4808
T	Signal transduction mechanisms	347	4.7665
U	Intracellular trafficking, secretion, and vesicular transport	34	0.4670
V	Defense mechanisms	135	1.8544
W	Extracellular structures	1	0.0137
Y	Nuclear structure	0	0.0000
Z	Cytoskeleton	0	0.0000
	**Total**	**7,280**	**100**

Table S4. List of predicted biosynthetic gene clusters from the genome sequence of strain THA-960. Biosynthetic gene clusters were predicted using antiSMASH (v8.0.4).

Region	Type	Chromosomal position	Most similar known cluster*	Similarity*
Region 1	NAPAA, CDPS	226,242-272,725		
Region 2	T3PKS	303,032-344,090	alkylresorcinol	100%
Region 3	siderophore	380,733-391,229		
Region 4	melanin	485,634-513,360		
Region 5	terpene	518,364-538,784		
Region 6	terpene	595,700-615,218		
Region 7	NRPS	661,615-711,876	coelichelin	90%
Region 8	thiopeptide, LAP, terpene	715,891-766,773		
Region 9	NRPS-like, NRPS	886,203-961,284	streptothricin	79%
Region 10	hglE-KS, T1PKS	1,256,791-1,306,587		
Region 11	NRPS	1,320,335-1,387,195		
Region 12	terpene	1,435,272-1,461,953	hopene	61%
Region 13	T1PKS	1,733,520-1,779,782		
Region 14	butyrolactone, T1PKS	1,787,859-1,833,264		
Region 15	terpene	1,857,982-1,876,476		
Region 16	RiPP-like	1,969,333-1,977,257		
Region 17	other, T1PKS, NRPS	2,078,970-2,181,347	himastatin	52%
Region 18	T2PKS, siderophore	2,269,264-2,339,974	fogacin A / fogacin B / fogacin C	56%
Region 19	arylpolyene, lanthipeptide-class-iii	3,234,085-3,275,224		
Region 20	NRPS, RiPP-like	3,813,497-3,856,347		
Region 21	NRPS-like, butyrolactone	4,144,512-4,185,534		
Region 22	butyrolactone, T1PKS, ladderane, phosphonate	4,748,577-4,834,386	amycomicin	87%

Region 23	siderophore	5,268,341-5,280,122	desferrioxamin B	100%
Region 24	NRPS, T1PKS, betalactone	6,864,131-6,936,276		
Region 25	T1PKS	6,969,453-7,014,003		
Region 26	NRPS-like, NRPS	7,133,414-7,200,418	JBIR-126 (tambromycin)	100%
Region 27	terpene	7,329,139-7,348,033	avermitilol	100%
Region 28	T2PKS	7,522,869-7,595,411	spore pigment	66%
Region 29	lanthipeptide-class-iii	7,703,944-7,726,580	SapB	100%
Region 30	NRPS, NRPS-like, terpene	7,961,535-8,031,493	carotenoid	63%
Region 31	lanthipeptide-class-iv	8,105,524-8,128,208	venezuelin	100%

*Only hits with similarity of 50% or greater were shown in the table.

Table S5. List of *S. virginiae* genomes available from NCBI genome database and average nucleotide identity (ANI) match details. Comparisons were performed using OrthoANI (v1.40) as described in STAR Methods.

		Best match type-strain*				
**Assembly Accession**	**Strain**	**Type assembly**	**Organism name**	**ANI**	**Assembly coverage**	**Type assembly coverage**
GCF_000716685.1	NRRL B-8091	GCA_014648615.1	*S. nojiriensis*	93.28%	73.08%	74.64%
GCF_000720455.1	NRRL ISP-5094	GCA_016755835.1	*S. virginiae*	96.55%	88.91%	84.12%
GCF_001270565.1	NRRL B-1447	GCA_014650695.1	*S. avidinii*	94.40%	76.72%	79.23%
GCF_014648795.1	JCM 4019	GCA_000720455.1	*S. virginiae*	96.53%	86.95%	89.19%
GCF_016755835.1	NBRC 15873	GCA_000720455.1	*S. virginiae*	96.55%	84.11%	88.90%
GCF_017876235.1	DSM 40803	GCA_000720455.1	*S. virginiae*	96.54%	86.48%	89.29%
GCF_026340475.1	NBC_01311	GCA_017639205.1	*S. nojiriensis*	93.32%	73.95%	74.66%
GCF_026341715.1	NBC_00640	GCA_017639205.1	*S. nojiriensis*	93.93%	82.35%	67.47%
GCF_026342285.1	NBC_00276	GCA_017639205.1	*S. nojiriensis*	93.85%	78.52%	70.12%
GCF_026342475.1	NBC_00227	GCA_016755855.1	*S. nojiriensis*	93.34%	71.78%	74.09%

*Data for the best match type-strain were obtained from NCBI genome database (accessed on January 10, 2024).

Table S6. Computational evaluation of amycomicin binding interfaces including structural confidence, interaction profiling, and predicted binding affinity using AlphaFold3.

UniProt Accession	A*	B*	C*	D*	E*	F*	G*	H*	I*	J*	K*	L*	M*	N*
A0A0X9W3U6	0.71	0.78	89.44	91.34	87.09	85.73	6.04	4.50	4.03	2.95	1	3	3	-6.77
A5HBL2	0.90	0.87	89.33	96.03	77.34	73.68	6.07	3.70	8.13	6.25	7	5	12	-9.47
P00807	0.90	0.88	92.21	98.40	81.08	78.80	6.20	2.40	5.82	4.60	8	4	7	-8.86
P0A0H3	0.90	0.90	90.76	96.28	77.76	73.14	5.39	3.20	5.11	3.85	2	10	10	-8.15
P0C1U9	0.62	0.86	88.96	94.05	66.98	59.85	17.21	20.30	8.58	7.45	3	5	6	-7.25
P13978	0.91	0.75	93.17	98.02	67.40	61.70	5.07	2.60	10.94	9.70	2	5	6	-8.04
P20831	0.60	0.82	80.38	89.49	52.70	44.75	18.78	24.40	9.88	9.10	1	0	2	-7.05
P41368	0.82	0.73	87.87	91.15	57.13	46.73	12.22	10.80	13.67	11.95	2	3	7	-9.78
P47768	0.89	0.60	85.02	88.79	38.29	22.30	9.41	7.20	18.52	17.35	3	2	7	-8.61
P61058	0.76	0.36	85.78	89.72	N/A**	N/A	9.39	6.30	N/A	N/A	N/A	N/A	N/A	-5.35
P66334	0.84	0.82	90.98	94.54	76.41	75.15	5.41	3.10	3.81	3.15	0	2	2	-6.46
P68790	0.69	0.75	86.57	92.54	61.24	54.09	13.61	15.50	13.22	11.80	5	6	11	-9.44
Q2G0E0	0.79	0.86	89.71	93.32	78.26	75.56	7.26	7.50	6.57	6.20	0	3	4	-6.83
Q2G2M2	0.57	0.90	76.24	80.69	76.71	75.98	16.45	17.80	8.26	8.05	4	5	7	-9.37
Q2YWY4	0.43	0.72	70.30	77.63	59.08	59.02	19.36	22.90	7.16	7.15	0	4	3	-7.46
Q2YYP6	0.79	0.24	84.29	87.90	25.79	6.50	9.20	5.20	23.15	23.15	0	2	2	-7.26
Q6G9K5	0.65	0.84	76.91	85.98	69.54	72.01	15.58	16.90	9.00	7.10	4	9	13	-9.65
Q6GAU3	0.95	0.91	95.95	98.08	81.90	81.51	3.20	2.20	5.99	4.55	5	8	14	-9.6
Q6GD85	0.68	0.86	81.14	87.99	75.96	77.50	14.88	17.00	7.13	5.75	3	8	8	-9.48
Q7A2Q1	0.72	0.88	89.50	93.20	78.57	76.65	9.08	6.30	5.97	5.25	1	4	4	-7.33
Q8NXR5	0.69	0.77	86.58	90.93	74.02	69.79	11.12	9.30	8.72	7.70	2	7	7	-7.66
Q99SZ7	0.77	0.90	83.83	87.98	80.05	78.88	10.22	8.10	5.59	5.45	3	6	9	-9.67
Q9RDT3	0.59	0.79	78.66	81.84	64.40	61.73	15.25	15.20	10.20	8.60	6	9	10	-9.85
Q9RDT5	0.68	0.77	83.61	89.79	67.55	64.37	9.52	9.30	7.81	7.05	3	0	3	-7.18

*A: Predicted Template Modeling score for the entire complex (ranges 0-1; the higher, the better), B: Interface pTM, a measure of the accuracy of

the interaction interface between the protein and the ligand, C-D: Mean and median for the Predicted Local Distance Difference Test (pLDDT) scores of all atoms, E-F: Mean and median for pLDDT scores specifically calculated for the residues and ligand atoms at the binding interface, G-H: Mean and median for Predicted Aligned Error (PAE) for the entire complex (the lower, the better), I-J: Mean and median for PAE scores focused on the relative positioning between the protein's binding site and the ligand, K-N: the number of hydrogen bonds, hydrophobic interactions, and general spatial contacts based on proximity thresholds predicted by PLIP, and M: the predicted Gibbs free energy of binding (ΔG) in kcal/mol predicted by PRODIGY-LIGAND (the lower, the better binding affinity). A-J were predicted by AF3.

[**]No data due to the lack of interface prediction.
